# Temperature Upshifts in Mammalian Cell Culture: A Suitable Strategy for Biosimilar Monoclonal Antibodies?

**DOI:** 10.3390/bioengineering10101149

**Published:** 2023-09-30

**Authors:** Lukas Marschall, Chitti Babu Gottimukkala, Biswajit Kayal, Veerabhadra Madurai Veeraraghavan, Samir Kumar Mandal, Suman Bandyopadhyay, Christoph Herwig

**Affiliations:** 1 TU Wien, Faculty of Technical Chemistry, Research Unit Biochemical Engineering, Gumpendorferstrasse 1a, 1060 Vienna, Austria; 2 Körber Pharma Austria GmbH, Mariahilfer Straße 88A/1/9, 1070 Vienna, Austria; 3 Dr. Reddy’s Laboratories Ltd., Biologics, Survey No. 47, Bachupally, Hyderabad 500090, India; chittibabugottimukkala@drreddys.com (C.B.G.); biswajitkayal@drreddys.com (B.K.); veerabhadra.mv@drreddys.com (V.M.V.); samirkm@drreddys.com (S.K.M.); sumanbandyopadhyay@drreddys.com (S.B.)

**Keywords:** biosimilar, mammalian cell culture, temperature downshift, scale-up

## Abstract

Temperature downshifts are the gold standard when setting up control strategies for mammalian cell culture processes. These shifts are performed to prolong production phases and attain heightened levels of productivity. For the development of biosimilars, however, the bottleneck is in achieving a prespecified product quality. In a late-stage development project, we investigated the impact of temperature shifts and other process parameters with the aim of optimizing the glycosylation profile of a monoclonal antibody (mAb). We applied a design of experiments approach on a 3 L scale. The optimal glycosylation profile was achieved when performing a temperature upshift from 35.8 °C to 37 °C. Total afucosylated glycan (TAF) decreased by 1.2%, and galactosylated glycan species (GAL) increased by up to 4.5%. The optimized control strategy was then successfully taken to the manufacturing scale (1000 L). By testing two sets of set points at the manufacturing scale, we demonstrated that the statistical models predicting TAF and GAL trained with small-scale data are representative of the manufacturing scale. We hope this study encourages researchers to widen the screening ranges in process development and investigate whether temperature upshifts are also beneficial for other mAbs.

## 1. Introduction

Temperature shifts are a common part of control strategies in cell culture monoclonal antibody (mAb) fed-batch processes [1]. In the published literature, temperature shifts are mostly performed from a higher set point to a lower set point at a defined point in time [2,3]. This is performed to promote cell growth in the beginning until a suitable cell density is reached. The temperature is reduced to maintain high viability and achieve higher specific productivities [2,4]. For these reasons, temperature downshifts have become part of platform strategies for cell culture processes. Masterton and Smales provided an extensive review of the impact of temperature on the cultivation of mammalian cell lines [1]. However, when developing biosimilar products or follow-on biologics, the similarity to the reference product needs to be demonstrated [5,6]. According to section 351(k) of the Public Health Service Act, a biosimilar is a biological product highly similar to the originator product in terms of safety, purity, and potency [7]. The structure of N-glycans is important for the biological activity of glycosylated biopharmaceuticals [8] and is, therefore, relevant when demonstrating biosimilarity. Achieving the desired glycan structure is often the bottleneck in the development of mAbs. Although they are beneficial to specific productivities, temperature downshifts might not be necessarily beneficial for achieving the desired glycosylation profile. The impact of temperature downshifts on quality attributes has been assessed in the scientific literature [9,10,11]. However, to our knowledge, the impact of temperature upshifts has not been discussed to date.

Because the idea behind the temperature downshift is driven by biological considerations and observed positive effects on the metabolism of cells [1], discussions on process development have mostly focused on the degree [1,9,10] and timing of temperature downshifts [3,12] rather than asking the question as to whether performing the downshift or even performing an upshift might be beneficial to product quality.

In late-stage process development, functional relationships between process parameters and quality attributes are usually investigated on a small scale [13]. The functional relationships are described as mathematical models (e.g., ordinary least-squares [OLS] regression), which are then consequently used to set up a control strategy for the manufacturing scale (in terms of set points, proven acceptable ranges, and design spaces) [14]. Regulatory authorities state that small-scale models must be representative of the manufacturing scale and emphasize the importance of understanding potential differences between the scales [15,16,17]. Therefore, a typical step in late-stage development is the qualification of the scale-down model, in which the representativeness of the scale-down model for the manufacturing scale is assessed. An approach most commonly followed is to statistically compare a group of runs from a manufacturing scale with a group of runs at a small scale performed at set-point conditions [18,19,20]. Various statistical techniques have been used for scale-down model qualification [21]. A disadvantage of these approaches is that they compare the scales only at set-point conditions and do not provide any insight as to whether the functional relationships between process parameters and quality attributes are the same. However, when setting up control strategies, it is important to determine whether a change in process parameter settings induces a similar change in product quality across the scales. This issue can be overcome only by purposefully introducing variance in process parameters in both scales. This involves changing a set-point of a process parameter from A to B in both scales and then comparing the change in quality attributes and performance in both scales. Such a comparison can either be conducted in one-factor-at-a-time studies or using design of experiment approaches. However, it is not economically feasible to run experiments at a manufacturing scale. The scientific literature contains studies that discuss the scale-up of mammalian cell culture processes. However, most of these studies rely on manufacturing data from a single set-point condition and do not include data from at least two different set points at a manufacturing scale [10,13,20,22,23]. Therefore, comparing these scales at set-point conditions is often just part of a risk-based approach, including process technological reasoning to underpin the assumption of similar effects across scales. For the reasons discussed, it is rare to have data available to support this claim, and as far as we are aware, there are no results in the scientific literature discussing the similarity of effects.

To our knowledge, we are the first to discuss the positive impact of a temperature upshift on product quality and titer of a mAb produced in a CHO fed-batch process. In addition, this report presents data supporting the claim that the effects of process parameters on quality attributes found on a small scale are the same at the manufacturing scale.

Within this contribution, we present the results of an experimental study performed in late-stage process development for the optimization of a mAb production process in CHO cells. We demonstrate that temperature upshifts can be a beneficial tool for optimizing the quality profile of a biosimilar product toward its originator product. In addition, we discuss the impact of the temperature upshift on process performance. We apply this temperature shift to the manufacturing scale and experimentally verify that the statistical models for quality attributes derived from the small scale are applicable to the manufacturing scale. Finally, we discuss the impact of the studied parameters and the temperature shift specifically on physiological rates and provide an outlook for potential follow-up studies.

## 2. Materials and Methods

### 2.1. Cell Line and Product

A stable CHO-S cell line expressing a therapeutic mAb of the IgG1 subtype was used in this study. The parent cell line used was a suspension-adapted CHO-S cell line obtained from GIBCO Life Technologies, Inc. (Rockville, MD, USA, Catalog No. 11619012). This cell line was developed by a US-based commercial vendor using multiple rounds of retroviral transduction of antibody-heavy and light chain-containing expression vectors. The cell bank was manufactured by the vendor and was characterized as per regulatory guidelines at Bioreliance (Rockville, MD, USA). These data are proprietary and cannot provided.

### 2.2. Process Description

All design of experiments (DoE) experiments were carried out in a scale-down model production bioreactor stage operated in a fed-batch mode with a working volume of 3 L (Sartorius). The inoculum expansion was performed over six passages. For the first passage, a thawed cryo vial was transferred into a 125 mL spinner bottle with 35 mL medium at an initial viable cell density of 0.2 × 10^6^ cells/mL. The cells were grown to a final viable cell density (VCD) of more than 0.8 × 10^6^ cells/mL. Consequently, three passages were performed in 500 mL spinner bottles with 180 mL medium at an initial viable cell density of 0.2 × 10^6^ cells/mL. In these stages, the cells were grown to a VCD of more than 1.3 × 10^6^ cells/mL. The fifth passage was performed in a 2000 mL spinner bottle with 900 mL medium at an initial viable cell density of 0.3 × 10^6^ cells/mL and 5% CO_2_. The cells were grown to a final VCD of more than 1.4 × 10^6^ cells/mL. All spinner stages were performed at 37 °C and 100 rpm. The last seed step was performed in a 6.5 L bioreactor with 5 L medium. We used the seed bioreactor stage to inoculate the production bioreactor stage (6.5 L glass bioreactor) with an initial volume of 3 L. The cells were grown to a final VCD of more than 2.4 × 10^6^ cells/mL. The initial volume was reduced accordingly, dependent on the volume of the feed to be added. The seed bioreactor and production bioreactor were inoculated to initial viable cell densities of 0.3 × 10^6^ cells/mL and 0.75 × 10^6^ cells/mL, and the stirring speeds were set to 100 rpm. In the production bioreactor, we added feed based on predefined integrated viable cell density (IVCD) values. Chemically defined PowerCHO^TM^ 2 medium, commercially available from Lonza (Walkersville, MD, USA), served as the basal medium for both the seed and production stages. Additionally, a 4× concentrated version of the same basal medium was used as a feed in all applicable experiments. A temperature shift was performed at the time point of feed addition. The temperature before the shift was 37 °C, and the temperature after the shift was 35 °C. The pH was controlled using CO_2_ sparging. The manufacturing scale production bioreactor was carried out in 1000 L stainless-steel bioreactors (Bioengineering). The seed train consisted of three spinner stages and three seed bioreactor stages.

### 2.3. Analytics

The bioreactors were sampled every day for routine analysis. We analyzed each sample for pH and dissolved gases in a blood gas analyzer (BGA) (Rapid Lab 348, Siemens Healthcare Diagnostics Inc., Tarrytown, NY, USA). If the difference between online pH (in the bioreactor) and offline pH (measured by BGA) was greater than ±0.05 units, the online pH probe in the bioreactor was recalibrated to the offline pH value. We measured the viable cell density and viability of cells using the Trypan blue exclusion method with a hemocytometer. The culture osmolality was measured using an osmometer (Model 2020 Osmometer, Advanced Instruments, Norwood, MA, USA). The concentrations of the metabolites (glutamine, glutamate, glucose, lactate, ammonia, sodium, and potassium) in the sample were analyzed using a Cedex Bio HT Analyzer (Roche Diagnostics GmbH, Mannheim, Germany). The harvest broth was analyzed for titer and glycopatterns. Titers were measured from a centrifuged harvest sample. The glycosylation profile was analyzed after a capture chromatography step in neutralized eluate.

The glycopattern assay was a multistep procedure that involved the enzymatic removal of glycans, labeling the isolated glycans with the fluorescent compound 2-aminobenzamide, and the separation and quantitation of individual species using amide chromatography with a high-performance liquid chromatography system (Waters Corporation, Milford, MA, USA) equipped with a fluorescence detector. Sample detection was achieved by sample excitation at 360 nm and emission detection at 425 nm.

### 2.4. Experimental Design and Available Data

We planned the experimental design in JMP 14.0 (SAS Institute GmbH, Heidelberg, Germany). We used the coordinate exchange algorithm as implemented in JMP to create the design. The I-optimality criterion was used to create a design that minimizes the average prediction variance. Ten thousand starts were carried out. To reduce the likelihood of overlooking effects in the screening range with respect to the responses, the aliasing structure and correlation structure of the DoE were assessed, and power analysis was performed. The required number of DoE runs was estimated to achieve at least 80% power to not overlook practically relevant changes in the responses. As practically relevant changes, the optimization targets, as discussed in the “Results” section, were used.

In total, we performed 60 DoE runs on a 3 L scale. The statistical models derived from these runs were used to find the optimal process parameter set points. The impact of the process parameter change was assessed on a 3 L scale and a 1000 L scale. On the 1000 L scale, 11 runs with initial set points and two runs with optimized set points were performed. On the 3 L scale, 15 runs with initial set points and 16 runs with optimized set points were performed.

### 2.5. DoE Analysis

OLS regression with fixed effects was used for statistical analysis. The factor matrix was scaled and centered according to Goos and Jones [24]. All displayed regression coefficients are the ones computed for centered and scaled data.

The block factors were modeled as fixed effects. In cases in which at least one block was found to be statistically significant, all blocks were automatically included in the model. The blocks were deviation-coded before the analysis [25]. By doing so, the model intercept represents the mean over all blocks. If blocking effects were active, they were set to 0 for the prediction plots. In that way, the mean of all blocks was used for predictions. This was performed as a block cannot be repeated and, therefore, should not be used for the prediction of future blocks. Future blocks are assumed to be part of the same population of blocks as the ones performed. Therefore, the best estimate for future blocks is the mean of the already observed blocks. The response variables were mean-centred prior to analysis.

We used the stepwise algorithm, with forward and backward steps (mixed mode), as implemented in PAS-X Savvy (2023). The *p*-value of the partial *t* statistic was chosen as the criterion for including and excluding factors. We chose a threshold-in value of 0.20 and a threshold-out value of 0.05 for each regressor. A strong heredity principle was followed (i.e., if a two-factor interaction was included in the model, the main effects of both factors involved in the interaction were included in the model as well, even if the main effects were not significant with the chosen threshold).

To ensure model adequacy, we performed a thorough analysis of the model residuals to check whether any of the assumptions for regression analysis were violated (i.e., the model errors were statistically independent, of constant variance, and normally distributed). In the reported study, the following metrics were used to evaluate the fitted OLS models: *R*^2^, adjusted *R*^2^ (*R*^2^_adj_), and leave-one-out cross-validated *R*^2^ (*Q*^2^). The root mean squared error of the model was compared with the standard deviation of historic runs at set-point conditions.

### 2.6. Calculation of Physiological Rates

Volumetric and specific rates were calculated as described in the literature [26]. Conversion rates were calculated according to Equation (1), and specific rates were calculated according to Equation (2):

(1) $$R_{i}=\frac{\partial m}{\partial t}-{\dot{V}}_{in}\times c_{in},$$

where *R_i_* is the conversion rate of component *i* [g/h], ${\dot{V}}_{in}$ is the inflow to the bioreactor [L/h], *c_in_* is the concentration of component *i* in feed [g/L], and $\frac{\partial m}{\partial t}$ is the mass change of component *i* in the bioreactor.

(2) $$q_{i}=\frac{R_{i}}{VCT},$$

where $q_{i}$ is the specific conversion rate [g/cell/h], and *VCT* is the total amount of viable cells [cells].

### 2.7. Calculation of Uncertainty of Difference in Means

We assessed the impact of the parameter optimization by comparing a set of runs at initial set-point conditions to a set of runs at optimized set-point conditions. The difference represents a change in means. To assess the uncertainty of this difference, we calculated a 95% confidence interval.

The 95% confidence interval for this difference in means was calculated according to Equation (3).

(3) μ* ±t1−α,   df  s1  2n1+s2  2n2

where µ* is the difference in means between the groups, α is the significance level, *df* is degrees of freedom, *s* is the standard of deviation for a group of runs, *n* is the number of observations in a group of runs, and t is the *t*-statistic from a Student’s *t*-distribution with *df* degrees of freedom.

The degrees of freedom (*df*) are calculated according to the Satterthwaite formula (Equation (4)) [27].

(4) $$df=\frac{{\left(\frac{s_{1}^{\;\;2}}{n_{1}}+\frac{s_{2}^{\;\;2}}{n_{2}}\right)}^{2}}{\frac{1}{n_{1\;}-1}\;{\left(\frac{s_{1}^{\;\;2}}{n_{1}}\right)}^{2}+\frac{1}{n_{2\;}-1}\;{\left(\frac{s_{2}^{\;\;2}}{n_{2}}\right)}^{2}}$$

where *S* is the standard deviation for a group of runs, and *n* is the number of observations in a group of runs.

## 3. Results

### 3.1. Description of DoE

We chose an I-optimal design with 60 runs to study six potentially critical process parameters: IVCD at feed addition (IVCD feed [×10^6^ cells*day/mL), pH set point (culture pH [−]), starting temperature (initial temp [°C]), temperature after the shift (temp after shift [°C]), dissolved oxygen set point (DO [%]), and the feed volume (feed volume [%*v*/*v*]). The design was planned in a way to resolve the main effects, two-factor interactions, and quadratic effects. We carried the design in five blocks of 12 runs. Because these share the same raw materials, seed train, and buffers, a blocking factor was introduced to account for their potential effects. Fourteen center-point runs were included to provide a measure of process stability and inherent variability. Four additional runs were replicated to obtain a better pure error estimate of the variance and to estimate the within-block variance. Three discrete levels were performed per factor, except for IVCD feed, for which a fourth level was performed. The screening ranges were defined based on the knowledge of the subject matter expert in a risk assessment. One bioreactor run did not work because of an operational error and was therefore excluded from the analysis.

**Figure 1:**
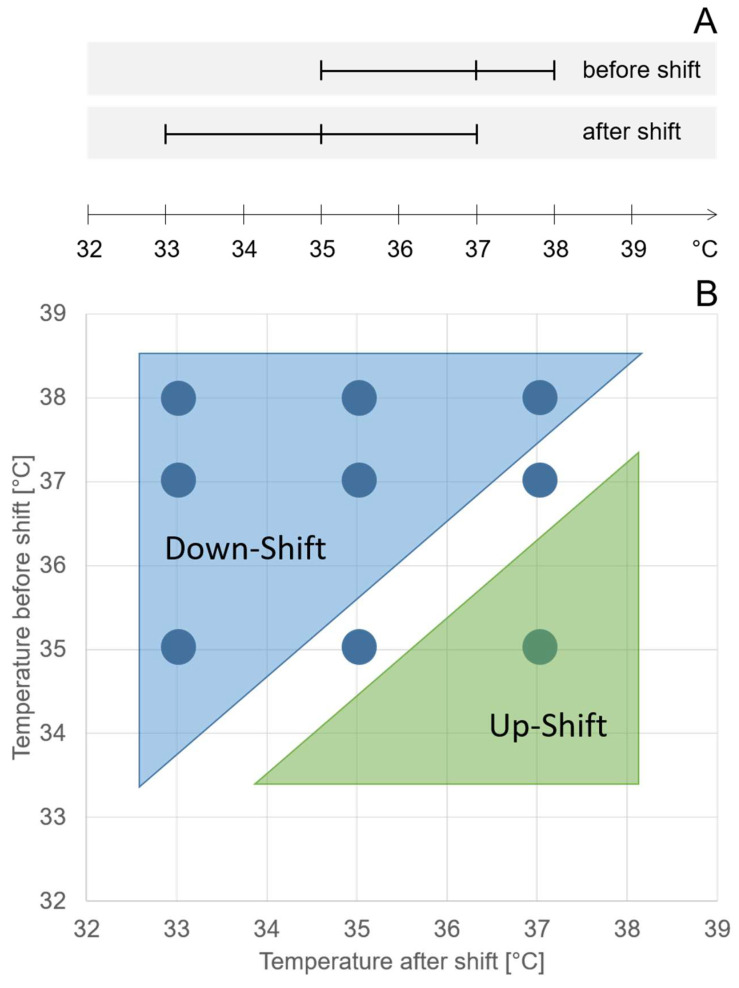
Visual depiction of the screening range for the factors temperature before and after the shift (**A**). The different factor combinations (shown as blue circles) resulted in six temperature downshifts, two constant temperatures, and one temperature upshift (**B**). The ranges for both temperatures were defined in a risk assessment. The full DoE table is given in the supplemental material.

Within the DoE, the investigated ranges of temperatures before and after shifts overlapped by 2 °C (Figure 1A), resulting in runs with temperature down-shifts, constant temperatures, and temperature up-shifts. We performed nine different combinations of temperature before and after the shift. Six-factor combinations resulted in temperature downshifts of different orders of magnitude (Figure 1B). Two-factor combinations resulted in a constant temperature, and one-factor combinations resulted in a temperature upshift (Figure 1B).

Total afucosylated glycan (TAF), which comprises high mannose species and afucosylated glycan species, galactosylated glycan species (GAL), and one performance indicator (titer) were modeled in separate OLS regression models. The value titers were normalized to the optimization target and are given in normalized units (nU). To assess the impact on process performance, features associated with culture viability and IVCD and physiological rates were modeled as well.

### 3.2. Optimization Goal and Strategy

**Figure 2:**
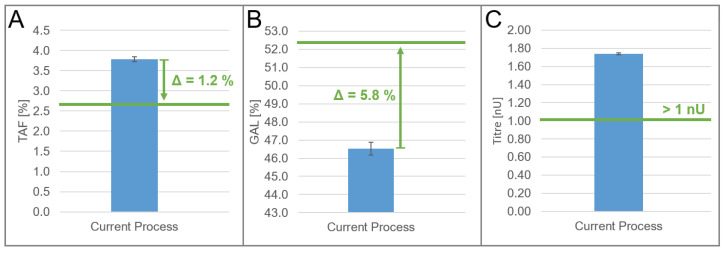
Quality profile and titer values at set-point conditions of the current process were calculated from 11 manufacturing runs. The error bars show the standard error of the responses. The optimization target is shown in green. The change in response to achieve the optimization target is shown as a green arrow. The mean values of (**A**) TAF, (**B**) GAL, and (**C**) titer at current set points on the manufacturing scale.

The identified models of the DoE were used to optimize the quality attribute profile, TAF, and GAL of the product at a manufacturing scale. We defined the optimization target for the two quality attributes in a way that the quality profile of the biosimilar product under study matches one of the originator products. The optimization target for titers was driven by business considerations. TAF should be decreased by 1.2% (Figure 2A), whereas GAL should be increased by 5.8% (Figure 2B). The titer should remain at least higher than 1 nU (Figure 2C); that is, it should not decrease by more than 0.74 nU.

We observed offsets in TAF, GAL, and titer at set-point conditions between small and manufacturing scales. Because the DoE was performed on a small scale, this offset needed to be taken into account when using the models derived from the DoE to optimize the manufacturing process. We assumed that the factor effects would still be the same across scales. We calculated the optimization target used for the DoE models by adding the anticipated change described in Figure 2 to the mean prediction of the models at set-point conditions (Table 1).

### 3.3. DoE Evaluation and Discussion of Models

All models for quality attributes and performance indicators explained at least 80% of the variation in the responses (*R*^2^_adj._), even after being corrected for the number of predictors. For physiological rates, the models explained at least 60% of the variation. All models exhibited an adequate prediction precision according to leave-one-out cross-validation (*Q*^2^) in the same range of the observed *R*^2^_adj._ values. The check for normal distribution of the residuals did not indicate any gross pattern or deviation from the normality assumption. Model summary tables and plots are provided in the supplementary material.

### 3.4. Impact of Temperature Shifts on Quality and Titer

In this study, the largest impacts on quality attributes and titer were found to be caused by culture pH and temperature after the shift. TAF showed a strong quadratic relation to temperature after the shift and a weaker relation to culture pH (Figure S1, Supporting Information). The culture pH had the largest impact on GAL and titer and showed a quadratic behavior (Figures S2 and S3, Supporting Information).

Both temperature factors before and after the shift were active in the models for TAF, GAL, and product titer. For TAF, both factors were active as quadratic effects and interacted with each other, with the effect of temperature after the shift being dependent on the factor setting of temperature before the shift (and vice versa). For GAL, temperature before the shift was active only as a main effect, and temperature after the shift showed curvature as well. For product titers, both factors were active as quadratic effects.

**Figure 3:**
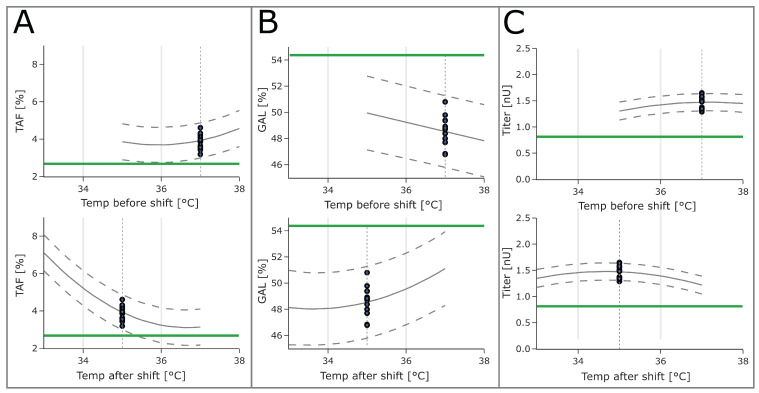
Univariate prediction plots for (**A**) TAF, (**B**) GAL, and (**C**) titer as a function of temperature before and after the shift. Each model parameter was varied within the screening range, whereas the other model parameters were kept at set-point conditions (grey dashed vertical line). Blocking effects were set to zero. The grey solid line shows the mean prediction at the respective parameter setting. The grey dashed lines represent the tolerance interval (95%/95%). The optimization target is shown as the green horizontal line. The DoE set-point runs are shown as blue dots.

Both temperatures showed large impacts on both glycopattern responses, with the temperature after the shift being the larger of the two (Figure 3). For product titers, the impact of both temperature factors was less pronounced. High temperature after the shift values moved the glycopattern responses closer to the targeted optimum. The same was true for lower temperatures before the shift. For both glycopattern responses, the optimization target could not be reached by varying only one of the temperatures. To reach the optimum, more than one parameter set point needed to be changed. For the titer, however, higher values were observed at a lower temperature after shift values and higher temperature before the shift.

**Figure 4:**
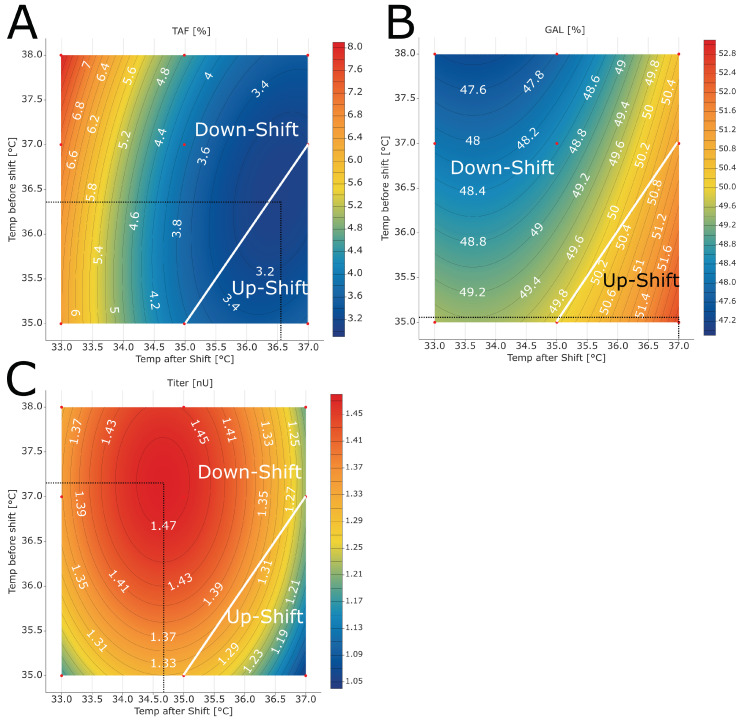
Contour plots of (**A**) TAF, (**B**) GAL, and (**C**) titer as a function of temperature before and after the shift. All other parameters were kept at set-point conditions. The black dashed lines mark the point closest to the optimization target. For the titer, all values are higher than the required minimum to be achieved, and the black dashed lines show the highest titer. The temperature conditions described by the white lines are runs without temperature shift (i.e., constant temperature). Above the white line, a temperature downshift was performed; below the white line, an upshift was performed. The red dots mark the factor combinations tested in the experiments. The red areas demark the temperature combinations leading to high TAF, GAL, and titer values, whereas the blue areas demark temperature combinations with lower values.

The fitted models revealed that temperature upshifts are beneficial to attain both glycopattern responses closer to the desired optimum (Figure 4A,B). Still, the optimum could not be reached by varying the two temperatures alone. A change in other process parameter set points was required as well. Previous investigations showed that temperature impacts the glycosylation profile of recombinant glycoproteins [10,11,27,28]. The changes in glycopattern might be associated with specific productivities and/or driven by temperature impacts on the activity of enzymes related to glycosylation [11,29]. However, comparison to other studies is difficult as the results are dependent on the used cell line, media, and control strategies (e.g., timing of temperature shifts). 

The optimum for product titer was found when performing a temperature downshift (Figure 4C). The highest titer was observed at a temperature downshift from 37.2 °C to 34.7 °C, which is in the typical range of downshifts commonly performed in mammalian cell culture [10]. The findings for titer are in line with the results reported by Kishishita et al., who also observed higher titers for shifts from 37 °C to 35 °C, compared with shifting from 37 °C to 33 °C [9]. This is in accordance with other reports in the scientific literature [10,11,30]. Lower titer values can be caused by either lower cell densities and/or lower specific production rates. We observed lower IVCD values for temperature upshifts (see discussion in Section 3.5), indicating that lower cell densities contributed to the decrease in the titer. In their report, Ahn et al. described higher specific productivities for temperatures lower than 37 °C [11]. They observed that the mRNA levels of the product are higher at lower temperatures and associated the increase in specific productivity with an increase in the transcription level. Due to the lack of timely resolved product concentration measurements, we could not verify this hypothesis.

### 3.5. Impact of Temperature Shifts on Viability and IVCD

**Figure 5:**
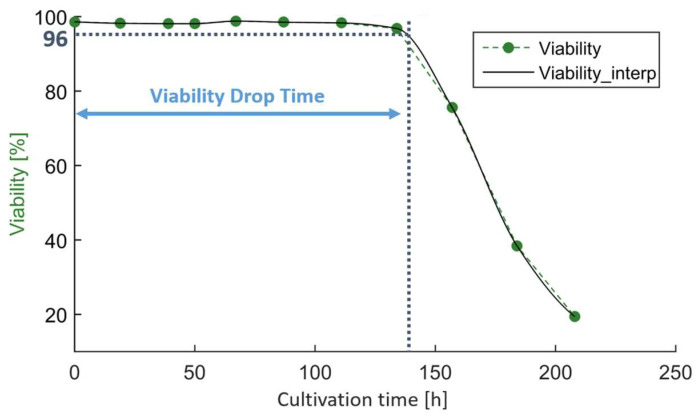
Viability drop time was determined by interpolating the viability time series and extracting the time at which the signal dropped below 96%.

As previously discussed, temperature downshifts are commonly performed to promote cell growth and maintain higher viabilities [2,3]. To put the current study into context, we also investigated the impact on cell growth and viability. As a feature for cell growth, we used the IVCD at the end of cell culture. As a feature for maintaining viability, we used the time until the viability dropped below 96%. We chose 96% so as not to falsely attribute a drop in viability due to analytical variability. Viability values were available in a timely resolution of 24 h. To identify the time point at which the viability dropped below the threshold, we interpolated the time series (piecewise cubic Hermite interpolating polynomial was used for interpolation, pchip as implemented in SciPy v1.11.2) (Figure 5).

**Figure 6:**
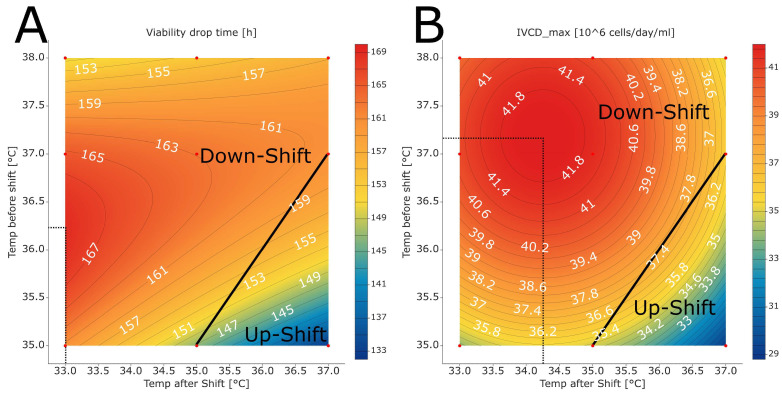
Contour plots of (**A**) viability drop time and (**B**) highest observed IVCD as a function of temperature before and after the shift. All other parameters were kept at set-point conditions. The grey dashed lines mark the optimum for the responses. The temperature conditions described by the black lines are conditions without temperature shift (i.e., constant temperature). Above the black line, a temperature downshift was performed; below the black line, an upshift was performed. The red dots mark the factor combinations tested in the experiments. The red areas demark the temperature combinations leading to high glycopattern and titer values, whereas the blue areas demark temperature combinations with lower values.

Temperatures before and after shifts were active as a two-factor interaction for the viability drop time, and a quadratic effect of temperature before the shift was observed as well (Figure S4, Supporting Information). The latest drop in viability was observed at a temperature downshift from 36.2 °C to 33.0 °C (Figure 6A). The lowest values were observed in the region of the temperature upshift. This finding is in line with the literature, as other contributions found that shifting the temperature from 37.0 °C to lower temperatures in CHO cell batch culture extends culture viability [2,10,31].

For IVCD at the end of the cell culture process, both temperatures before and after the shift were active as quadratic effects (Figure S5, Supporting Information). The highest IVCD value was observed at a temperature downshift from 37.2 °C to 34.3 °C (Figure 6B). The lowest values were observed in the region of the temperature upshift. The achievement of higher cell densities when performing temperature downshifts is in line with the findings in the available literature [10,11].

Our findings regarding the impact of temperature on culture viability and achieved cell densities fit well with the available scientific literature.

### 3.6. Application to Manufacturing Scale Optimization 

To optimize the glycopattern profile, we used the desirability profiling function as implemented in JMP. For each response, we defined a desirability function according to the optimization target given in Table 1. The overall desirability to be maximized is the geometric mean of the individual desirability functions [32]. The optimum of the glycopattern responses was achieved by decreasing the temperature before the shift from 37.0 °C to 35.8 °C and increasing the temperature after the shift from 35.0 °C to 37.0 °C, resulting in a temperature upshift. As described previously, the optimum could not be reached by solely varying the temperature before and after the shift. Other factors needed to be varied as well. Because this is a commercial process, we cannot provide these data set points for the other studied parameters. The changed temperature set points are summarised in Table 2. For the new set points, the model predicted a decrease in TAF by 1.1%, an increase in GAL by 4.7%, and a decrease in the titer by 0.27 nU.

The new set points for the optimized process were tested at the manufacturing scale and small scale. Two manufacturing runs with the temperature upshift process were compared with 11 manufacturing runs with the original set points. On the small scale, 16 runs with new set points (temperature upshift) were compared with 15 runs with the original set points. 

**Figure 7:**
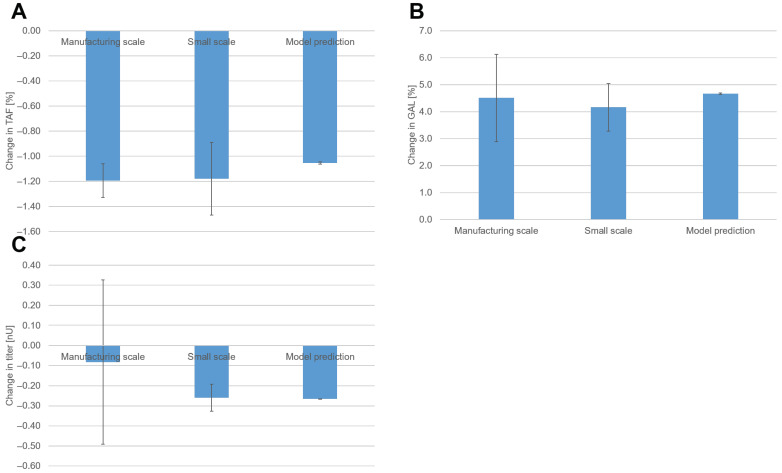
Change in (**A**) TAF, (**B**) GAL, and (**C**) titers before and after set-point optimization was assessed by calculating the difference in means of a group of runs with old set points and a group of runs with new set points. For manufacturing, 11 runs with the original set point and two runs with the temperature upshift process were available. For small scale, 15 runs with the original set point and 16 runs with the temperature upshift process were available. The error bars for the small scale and manufacturing scale represent the 95% confidence interval. For model prediction, the error bars are the root mean squared error divided by the square root of the number of DoE runs.

With the new set points, TAF decreased by 1.2% in both scales (Figure 7A). GAL increased by 4.5% on the manufacturing scale and by 4.2% on the small scale (Figure 7B). For TAF and GAL, the observed changes fit quite well with the predicted changes in the model. As discussed previously, we observed offsets in glycopatterns across scales. The results of the optimization showed that despite these offsets, the effects across scales were the same. Hence, the models for glycopatterns derived from the DoE performed on the small scale were also applicable to the manufacturing scale.

With the new set points, the titer decreased by 0.08 nU on the manufacturing scale and 0.26 nU on the small scale (Figure 7C). The 95% confidence interval for manufacturing titers had a relatively large range due to a limited number of degrees of freedom. Since this confidence interval encompassed zero, the observed value could not be deemed significantly different from zero at a 5% significance level.

For titers, the model accurately predicted the change on a small scale. On the manufacturing scale, however, the observed decrease in titers was not as high as predicted. These findings indicate that for titers, the small scale and the available statistical model are not representative of the manufacturing scale. Vodopivec et al. also observed differences in product titer during the scale-up of a CHO fed-batch cultivation from 1 to 1000 L [33]. Chaudhary et al. observed differences in titer between 2 L and 12 kL [34]. They identified higher gas entrance velocities on the manufacturing scale as the root cause of these differences. Another potential root cause might be inhomogeneities caused by increased mixing times on manufacturing scales [35]. Additional research is required to investigate the factors contributing to the lack of model representativeness on the manufacturing scale.

### 3.7. Discussion of Physiological Rates

The following conversion rates were calculated: specific growth rate, specific glucose uptake rate, and specific lactate formation/uptake rate. The cell culture process was divided into two phases: before the temperature shift and after the temperature shift. For both phases, the average values for the calculated conversion rates were calculated, resulting in six features that were consequently modeled as a function of the studied process parameters. The DoE runs performed on a small scale were used to model the six features. For the analysis of the physiological rates in the phase before the shift, we removed the three process parameters IVCD at feed addition, feed volume, and temperature before the shift as independent variables, as they were defined only for the phase after the shift. It must be stressed that available data on substrate, glucose, and lactate were subject to random noise due to sample handling and analytical variability. To ensure the accuracy and reliability of these data, we excluded from analysis certain values deemed too extreme and could not be accounted for by the process or by the natural limitations of mammalian cells, such as increases in glucose levels, although no feed was added. Still, the resulting rates are subject to noise, and the extracted features show relatively high variability.

#### 3.7.1. Lactate Formation/Uptake

Before the temperature shift, the specific rate of lactate formation is mainly driven by the process parameters culture pH and temperature (Figure S6, Supporting Information). We observed higher specific lactate formation at higher pH values and higher temperatures. No impact of DO was observed.

**Figure 8:**
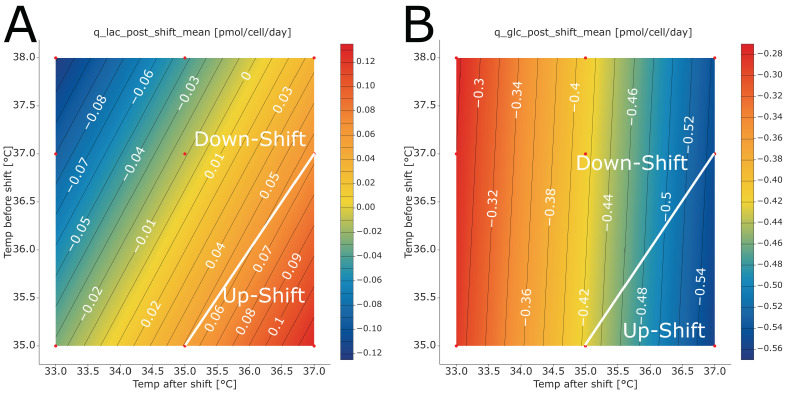
Contour plots of (**A**) specific lactate conversion rate after the temperature shift and (**B**) specific glucose uptake rate after the temperature shift as a function of temperature before and after the shift. All other parameters were kept at set-point (control) conditions. The temperature conditions described by the white lines are runs without temperature shift (i.e., constant temperature). Above the white line, a temperature downshift was performed; below the white line, an upshift was performed. The red dots mark the factor combinations tested in the experiments. The red areas demark temperature combinations, leading to high glycopattern and titer values, whereas the blue areas demark temperature combinations with lower values.

The largest impact on specific lactate formation/uptake rate after the shift was observed for pH (quadratic), temperature before the shift, and temperature after the shift (Figure S7, Supporting Information). Higher lactate uptake was observed for high pH values and low temperature after shift values. The model involves multiple two-factor interactions (2FIs). The two largest 2FIs were found for culture pH, with the time point of feed addition (which is equivalent to the time point of temperature shift) and temperature after the shift. The positive effect of culture pH on lactate formation becomes steeper at a higher temperature after shifts and at earlier time points of feed addition (Figure S8, Supporting Information). Interestingly, higher temperatures before the shift also favor a higher lactate uptake after the shift. This might be attributed to the fact that at higher initial temperatures, more lactate is also produced. This is in line with other reports in the scientific literature where higher specific lactate formation was observed at higher temperatures [11,30]. Compared with the phase before the shift (0.45 to 2.36 pmol/cell/day), the impact on lactate formation/uptake is considerably lower in the phase after the shift (−0.16 to 0.14 pmol/cell/day). However, the setting of the process parameters does still affect whether lactate is consumed or produced. In general, when shifting the temperature from lower to higher temperatures, we observed a tendency for lactate formation after the shift (Figure 8A). Conversely, we observed lactate uptake after the shift when shifting from higher to lower temperatures.

#### 3.7.2. Glucose Uptake

Culture pH was the only factor found to influence the specific rate of glucose uptake before the temperature shift (Figure S9, Supporting Information). The glucose uptake increased with increasing pH. The observation of higher glucose uptake rates at higher pH values is in line with recent findings in the literature [36]. After the temperature shift, culture pH was still found to have the largest impact on specific glucose uptake (Figure S10, Supporting Information). The second largest effect was temperature after the shift. The cells consume more glucose at higher pH and higher temperatures after shift values. Culture pH is involved in three two-factor interactions with the time point of feed addition, temperature before the shift and temperature after the shift (Figure S11, Supporting Information). However, the impact of these 2FIs is negligible compared with the size of the main effects. Because the impact of temperature before the shift and any 2FIs in which it is involved is considerably low, the direction of the temperature shift does not affect the specific glucose uptake after the shift. However, it is influenced by the temperature after the shift. Similarly, uptake rates were achieved for a temperature downshift from 38 °C to 37 °C and a temperature upshift from 35 °C to 37 °C.

#### 3.7.3. Specific Growth Rate

Culture pH and temperature show the largest impact on the specific growth rate before the temperature shift (Figure S12, Supporting Information). The specific growth rate increases with increasing pH and temperature values. It shows a quadratic dependency on culture pH and reaches a plateau of approximately 0.0295 [h-1].

After the temperature shift, the specific growth rate dropped from an average of 0.025 [h-1] to −0.0001 [h-1]. For some runs, we observed negative growth rates, which are attributed to the decrease in viable cell density in the phase after the shift. The impact of process parameters on the growth rate was considerably lower in the phase after the shift (Figure S13, Supporting Information). We found the largest effects on specific growth rates for the time point of feed addition and initial temperature. However, the effects were small compared with the effects on specific growth rates before the shift. Culture pH was found to be active in two 2FIs, with the time point of feed addition and the initial temperature (Figure S14, Supporting Information).

In general, in the phase before the shift, high specific growth rates were coupled with high specific glucose uptake (*R*^2^ = 0.629) and high specific lactate formation rates (*R*^2^ = 0.51) (Figure S15, Supporting Information). We observed high specific lactate formation rates for high specific uptake rates (*R*^2^ = 0.83). In the phase after the shift, the specific growth rate dropped almost to zero. Depending on the temperature setting after the shift, lactate metabolism was affected. Temperature downshifts triggered lactate uptake. Lactate formation was observed for temperature upshifts, although at a lower rate compared with the phase before the shift. The factor showing the largest impact on the investigated physiological rates was the culture pH and its 2FIs. The temperatures before and after the shift also played a role, but to a lesser extent compared with culture pH.

#### 3.7.4. Impact on Physiological Rates of Optimized Process Parameter Set Points

**Figure 9:**
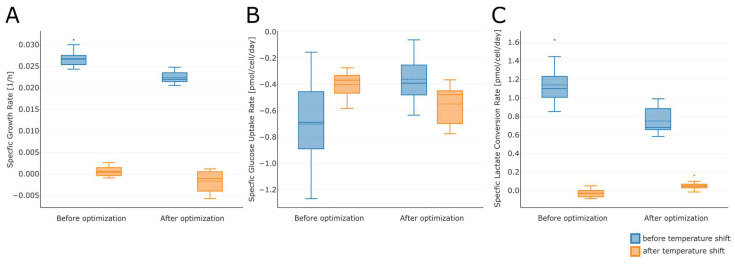
Comparison of (**A**) specific growth rate, (**B**) specific glucose uptake rate, and (**C**) specific lactate conversion rate before and after process optimization on the small scale. Fifteen runs with the original set point (control) and 15 runs with the temperature upshift process were available. The dashed lines represent the mean; the solid line in the middle of the box represents the median.

We compared the physiological rates of 16 small-scale runs after optimization (temperature upshift) to 15 small-scale runs before optimization. The comparison confirmed the predictions of the models. Lower specific growth rates were observed for lower initial temperatures, whereas hardly any impact on specific growth rates was observed after the temperature shift (Figure 9A). As in the DoE after the temperature shift, we observed negative growth rates on average, which are attributed to a decrease in viable cell density in the phase after the shift. The impact of process parameters on the growth rate was considerably lower in the phase after the temperature shift. The runs with a temperature upshift process showed lower specific glucose uptake rates before the shift and higher uptake rates after the shift compared with the original set points (Figure 9B). This was expected, as in the optimized process, the temperature before the shift was lower, but the temperature after the shift was higher compared with the original set point. Similarly, we observed lower specific lactate uptake rates before the shift for the optimized process. After the temperature shift, the specific lactate uptake rate was positive on average for the optimized process (Figure 9C), thus confirming the predictions of the statistical models. The time profile trends of viability, VCD, lactate, glucose, and pH are provided as supporting information (Figures S16–S20, Supporting Information).

## 4. Conclusions

Within this study, we showed that temperature upshifts were beneficial to achieve the desired glycosylation profile of a therapeutic mAb of IgG1 subtype produced in a mammalian cell culture process. We demonstrated that in the studied system, starting at lower temperatures and shifting to higher temperatures led to an optimized glycosylation profile. The improvement in quality went hand in hand with a decrease in product titer and lower cell densities. Other reports in the scientific literature also highlight that lowering the temperature has a positive impact on product titers [9,10,11,30]. However, as McHugh et al. showed in their study, the effects vary between cell lines and products [10]. The impact of temperature on the glycosylation profile of recombinant glycoproteins has been discussed in other reports as well [10,11,28,29]. However, the findings are dependent on the cell lines, products, and process conditions. Further studies on how temperature affects the glycosylation of recombinant proteins would be required.

During the planning of the experimental designs, we thoroughly discussed the screening ranges. The chosen ranges for temperature before the shift and after the shift resulted in factor combinations involving a temperature upshift. Initially, we planned to restrict the screening range in a way that only allows for temperature downshifts. However, we finally decided not to restrict the screening range for two reasons. First, allowing a wider range of process parameters increased the statistical power to identify potential effects. Second, not restricting the design space increased the process knowledge gained from the experiments. In our case, the process optimum for product quality would have been missed if temperature upshifts had been excluded pre-emptively from the design.

We could not assess the link between physiological rates and product quality, as it was not the initial purpose of the performed study. The purpose was to investigate the impact of process parameters on product quality. For that reason, the calculated rates are themselves a function of the process parameters and, therefore, cross-correlated with the studied quality attributes. In addition, timely resolved product data were not available. The product formation dynamics could not be assessed, as specific product rates could not be calculated. For future studies, we would recommend an analysis of timely resolved product amount, product quality, and amino acids to enable a thorough investigation of metabolic pathways. In their review, Reddy et al. highlighted the scarcity of studies that dynamically modeled the impact of temperature on metabolism and glycosylation [37]. To gain a deeper understanding of how lowering or increasing the temperature affects the glycosylation profile, we want to encourage systematic studies investigating the impact of temperature on metabolism and glycosylation using dynamic metabolic flux analysis.

Typically, for scale-down model qualifications, only manufacturing runs at one set-point condition are available, and scale-down models are considered to be comparable with the manufacturing scale if they yield similar results at set-point conditions. Within the study, a DoE was performed on a 3 L scale, and two different sets of set points were performed on the manufacturing scale (1000 L). This allowed the comparison of the effects of process parameters on quality attributes between scales. Although we observed an offset in the investigated quality attributes between the scales at set-point conditions, the anticipated change in the glycosylation profile on the manufacturing scale matched the predicted change from the small-scale model, thereby demonstrating that the assumption of similarity of effects of scales does apply to the studied quality attributes. We acknowledge that the two sets of set-point conditions performed in this study on the manufacturing scale do not allow for a proper statistical comparison of process parameter effects between scales. To conduct a more thorough data-driven comparison of scales, it would be necessary to include the scale as a factor in the DoE. This approach would allow modeling the process parameter effects individually for each scale and enable a proper comparison using analysis of variance. However, it is worth noting that performing multiple set-point conditions on the manufacturing scale, while desirable for robust analysis, may not be economically feasible under typical conditions.

For product titers, the fitted statistical model trained with small-scale data failed to accurately predict the change in manufacturing scale. In general, we observed higher titer values on manufacturing scales compared with small scales. Differences in titers between scales have already been observed elsewhere [33,34]. In order to understand the observed differences in our system, further studies are required to focus on the difference in titers between scales at set-point conditions first. As a subsequent step, we would test the hypothesis if the identified root causes for the offset are also responsible for the statistical model’s lack of representativeness at the manufacturing scale.

To achieve higher productivity in mammalian cell cultures, temperature downshifts have become entrenched in bioprocess development. The discussion mainly evolves around the degree and the timing of the temperature downshift. We demonstrated that looking in the other direction might prove to be beneficial for product quality.

## Figures and Tables

**Table 1:** Current glycopattern and titer values at set-point conditions in manufacturing scale. The standard error is given in parentheses. For small scale, the mean prediction of the DoE models at set-point conditions is given. The optimization targets for the small scale were calculated by adding the intended change to the prediction at set-point conditions.

	Performance at Set-Point Conditions		Optimization Target	
	Manufacturing Scale	Small Scale (Model Prediction)	Manufacturing Scale	Small Scale
TAF (%)	3.8 (0.06)	3.9	2.6	2.7
GAL (%)	46.5 (0.35)	48.5	52.3	54.4
Titer (nU)	1.74	1.47	1	0.73
Number of runs	11	60	-	-

**Table 2:** Parameter set points for temperature before and after optimization. The temperatures before and after the shift are given in °C. All other parameters are given in coded units. The values were scaled in a way that the screening range of the DoE lies in the interval of [−1, 1].

	Set Points before Optimization	Set Points after Optimization
Temperature before shift (°C)	37.0	35.8
Temperature after shift (°C)	35.0	37.0
IVCD feed (−)	−0.2	0.8
Culture pH (−)	0	−0.07
DO (−)	0	−0.8
Feed volume (−)	0	−1

## Data Availability

The data are not publicly available as this is a commercial process.

## Supplementary Materials

The following supporting information can be downloaded at https://www.mdpi.com/article/10.3390/bioengineering10101149/s1, Figure S1: Model summary for Glycopattern 1; Figure S2: Model summary for Glycopattern 2; Figure S3: Model summary for Titer; Figure S4: Model summary for the viability drop time; Figure S5: Model summary for IVCD_max; Figure S6: Model summary for the mean specific lactate conversion rate before the temperature shift; Figure S7: Model summary for the mean specific lactate conversion rate after the temperature shift; Figure S8: Interactions plot for the model describing the specific lactate conversion rate after the temperature shift; Figure S9: Model summary for the mean specific glucose uptake rate before the temperature shift; Figure S10: Model summary for the mean specific glucose uptake rate after the temperature shift; Figure S11: Interactions plot for the model describing the specific glucose uptake rate after the temperature shift; Figure S12: Model summary for the mean specific growth rate before the temperature shift; Figure S13: Model summary for the mean specific growth rate after the temperature shift; Figure S14: Interactions plot for the model describing the specific growth rate after the tempera-ture shift; Figure S15: Cross correlation of mean of physiological rates in the phase before temperature shift; Figure S16: Comparison of the viability trend over time before and after optimization; Figure S17: Comparison of the VCD trend over time before and after optimization; Figure S18: Comparison of the lactate trend over time before and after optimization; Figure S19: Comparison of the glucose trend over time before and after optimization; Figure S20: Comparison of the pH (measured online) trend over time before and after optimization; Table S1: DoE design table. Temperature before and after shift are given in original units. All other parameters are given in coded units.
