# Supplementary material for: Temperature Upshifts in Mammalian Cell Culture: A Suitable Strategy for Biosimilar Monoclonal Antibodies?
Source: Bioengineering (Basel). 2023 Sep 30;10(10):1149. doi: 10.3390/bioengineering10101149 (PMC10603922; doi:10.3390/bioengineering10101149)
Supplement: Supplementary file 1 [file bioengineering-10-01149-s001.zip › bioengineering-2597567-supplementary.pdf]

Table 1. DoE design table. Temperature before and after shift are given in original units. All other parameters are given in coded units.

Run	Temp after shift	Temp before shift	IVCD feed	Culture pH	DO	Feed volume	Block
1	35	37	-0.2	0	0	0	1
2	35	35	-1	-1	-1	1	1
3	33	38	-1	0	1	-1	1
4	33	35	1	-1	1	0	1
5	37	37	-1	-1	1	-1	1
6	35	37	-0.2	0	0	0	1
7	33	38	-0.2	-1	-1	-1	1
8	37	35	-1	1	0	-1	1
9	37	35	-1	1	0	-1	1
10	37	38	1	-1	0	1	1
11	35	38	-0.2	0	-1	1	1
12	35	37	-0.2	0	0	0	1
13	35	37	-0.2	0	0	0	2
14	33	37	-1	1	-1	0	2
15	37	35	-0.2	1	-1	1	2
16	37	38	-1	-1	0	0	2
17	37	37	1	1	1	0	2
18	35	37	-0.2	0	0	0	2
19	37	37	1	1	1	0	2
20	37	38	-0.2	1	1	-1	2
21	33	38	1	1	1	1	2
22	33	35	-1	-1	0	-1	2
23	37	35	1	0	1	-1	2
24	35	37	-0.2	0	0	0	2
25	35	35	-1	1	1	0	3
26	37	37	1	-1	-1	-1	3
27	33	38	-0.2	-1	1	1	3
28	33	38	1	1	0	-1	3
29	35	38	-1	1	0	1	3
30	33	35	1	0	-1	0	3
31	35	37	-0.2	0	0	0	3
32	35	37	-0.2	0	0	0	3
33	37	35	-0.2	-1	-1	0	3
34	37	35	-0.2	-1	-1	0	3
35	35	37	-0.2	0	0	0	3
36	35	35	1	-1	1	1	3
37	33	37	-0.2	1	1	-1	4
38	35	37	-0.2	0	0	0	4
39	33	37	1	-1	0	1	4
40	33	35	-0.2	1	0	1	4
41	37	38	-1	0	-1	-1	4
42	35	38	1	-1	1	-1	4

43	35	37	-0.2	0	0	-7	4
44	35	35	1	1	-1	-1	4
45	33	37	1	-1	0	1	4
46	37	37	-1	0	1	1	4
47	35	37	-0.2	0	0	0	4
48	37	38	1	1	-1	0	4
49	35	37	2.5	0	0	0	5
50	35	37	-0.2	0	0	0	5
51	35	37	-0.2	1	0	0	5
52	37	38	1	0	1	0	5
53	37	35	2.5	1	0	1	5
54	35	35	2.5	-1	-1	-1	5
55	33	38	2.5	0	-1	1	5
56	35	37	-0.2	0	0	0	5
57	33	37	2.5	1	1	-1	5
58	35	37	1	1	-1	1	5
59	33	35	-1	-1	1	1	5

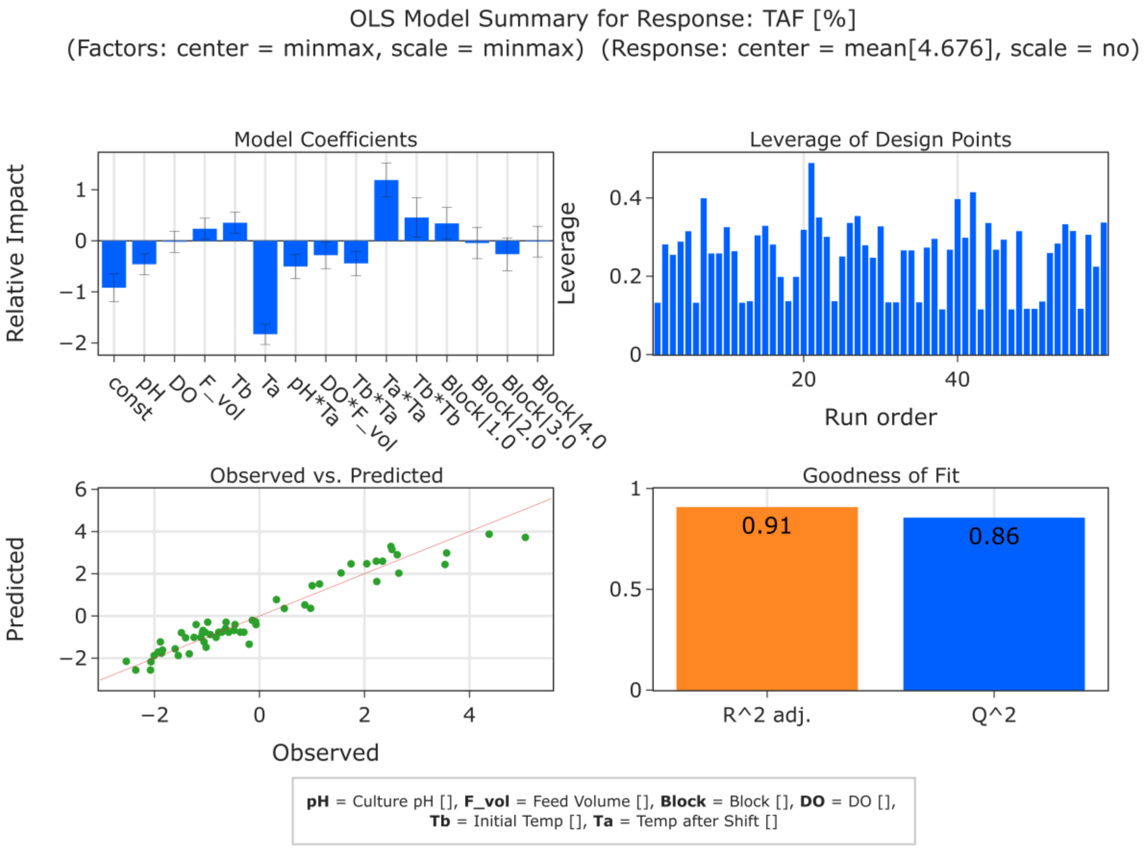

Figure S1. Model summary for Glycopattern 1. In the subplot “Model coefficients”, the scaled and centered model coefficients are shown. Main effects can be interpreted as the change of the response variable (in original units) within half the screening range of the respective factor. The coefficients of the fixed effects are depicted in blue. Displayed intervals represent the 95% confidence interval. The second subplot shows the leverage of each individual run in the model. No individual run shows particularly high leverage. In the third subplot, the observed vs predicted values by the model are displayed. In the last subplot, "Goodness of Fit", the R2 adj. and Q2 values from the model are displayed.

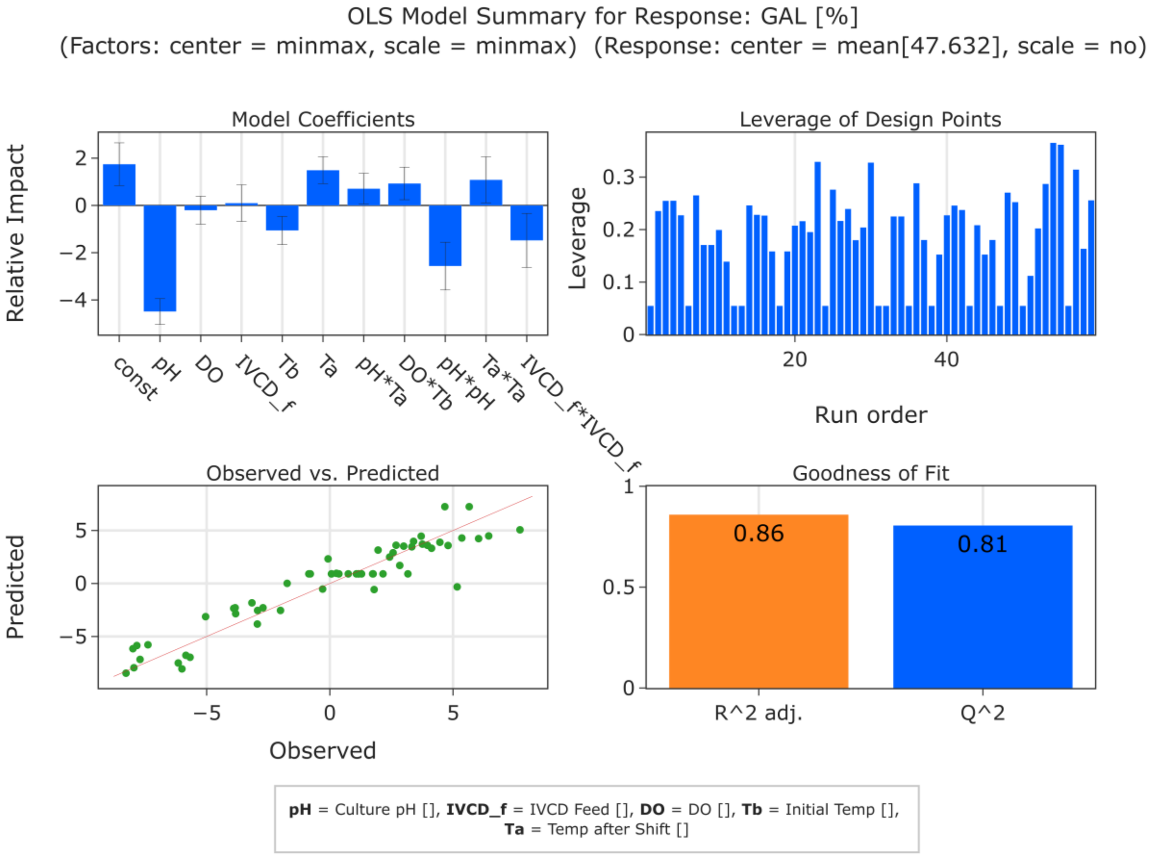

Figure S2. Model summary for Glycopattern 2. In the subplot “Model coefficients”, the scaled and centered model coefficients are shown. Main effects can be interpreted as the change of the response variable (in original units) within half the screening range of the respective factor. The coefficients of the fixed effects are depicted in blue. Displayed intervals represent the 95% confidence interval. The second subplot shows the leverage of each individual run in the model. No individual run shows particularly high leverage. In the third subplot, the observed vs predicted values by the model are displayed. In the last subplot, "Goodness of Fit", the R2 adj. and Q2 values from the model are displayed.

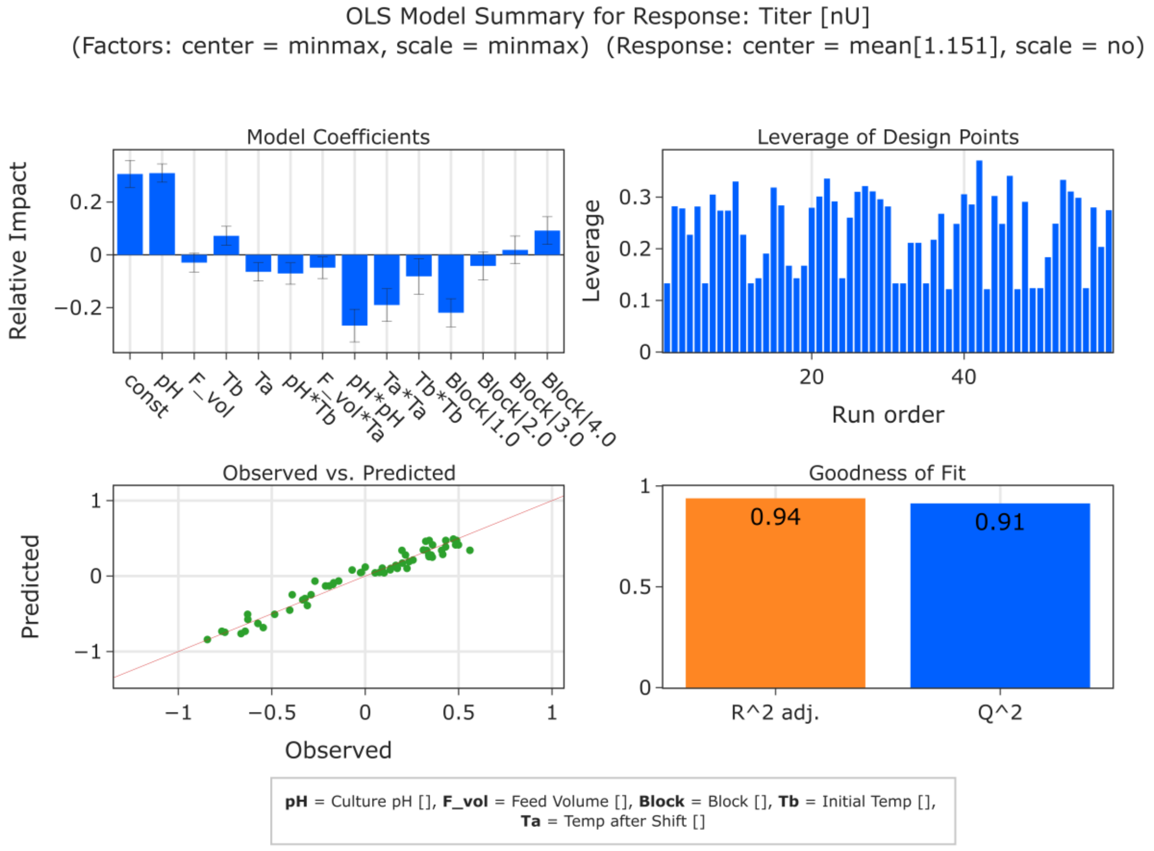

Figure S3. Model summary for Titer. In the subplot "Model coefficients", the scaled and centered model coefficients are shown. Main effects can be interpreted as the change of the response variable (in original units) within half the screening range of the respective factor. The coefficients of the fixed effects are depicted in blue. Displayed intervals represent the 95% confidence interval. The second subplot shows the leverage of each individual run in the model. No individual run shows particularly high leverage. In the third subplot, the observed vs predicted values by the model are displayed. In the last subplot, "Goodness of Fit", the R2 adj. and Q2 values from the model are displayed.

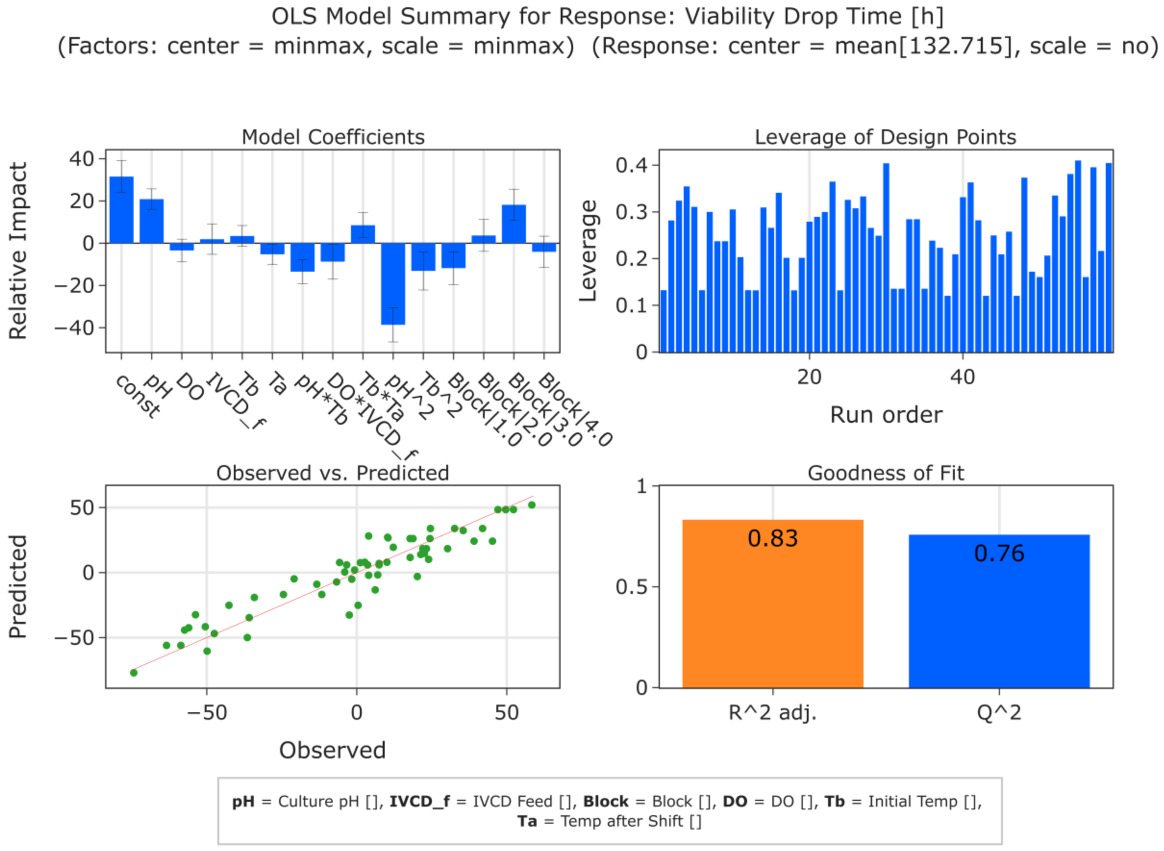

Figure S4. Model summary for the viability drop time. In the subplot “Model coefficients”, the scaled and centered model coefficients are shown. Main effects can be interpreted as the change of the response variable (in original units) within half the screening range of the respective factor. The coefficients of the fixed effects are depicted in blue. Displayed intervals represent the 95% confidence interval. The second subplot shows the leverage of each individual run in the model. No individual run shows particularly high leverage. In the third subplot, the observed vs predicted values by the model are displayed. In the last subplot, "Goodness of Fit", the R2 adj. and Q2 values from the model are displayed.

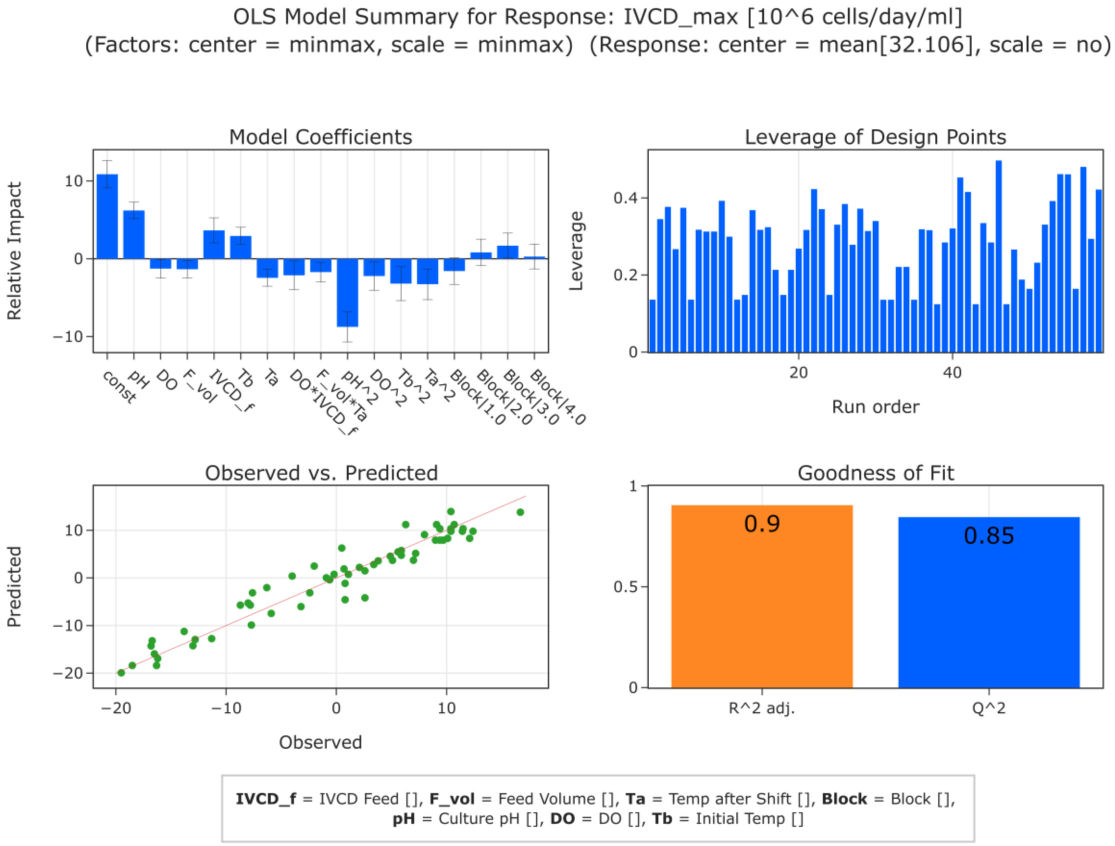

Figure S5. Model summary for IVCD_max. In the subplot "Model coefficients", the scaled and centered model coefficients are shown. Main effects can be interpreted as the change of the response variable (in original units) within half the screening range of the respective factor. The coefficients of the fixed effects are depicted in blue. Displayed intervals represent the 95% confidence interval. The second subplot shows the leverage of each individual run in the model. No individual run shows particularly high leverage. In the third subplot, the observed vs predicted values by the model are displayed. In the last subplot, "Goodness of Fit", the R2 adj. and Q2 values from the model are displayed.

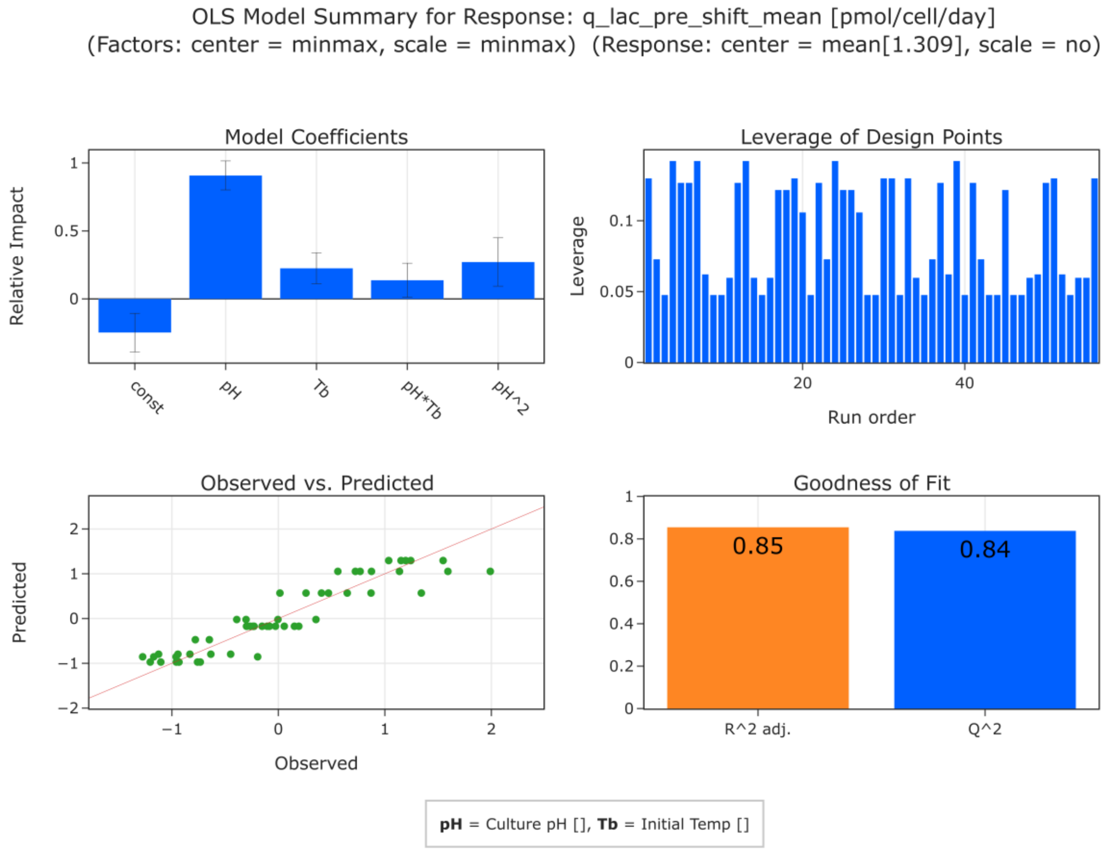

Figure S6. Model summary for the mean specific lactate conversion rate before the temperature shift. In the subplot "Model coefficients", the scaled and centered model coefficients are shown. Main effects can be interpreted as the change of the response variable (in original units) within half the screening range of the respective factor. The coefficients of the fixed effects are depicted in blue. Displayed intervals represent the 95% confidence interval. The second subplot shows the leverage of each individual run in the model. No individual run shows particularly high leverage. In the third subplot, the observed vs predicted values by the model are displayed. In the last subplot, "Goodness of Fit", the R2 adj. and Q2 values from the model are displayed.

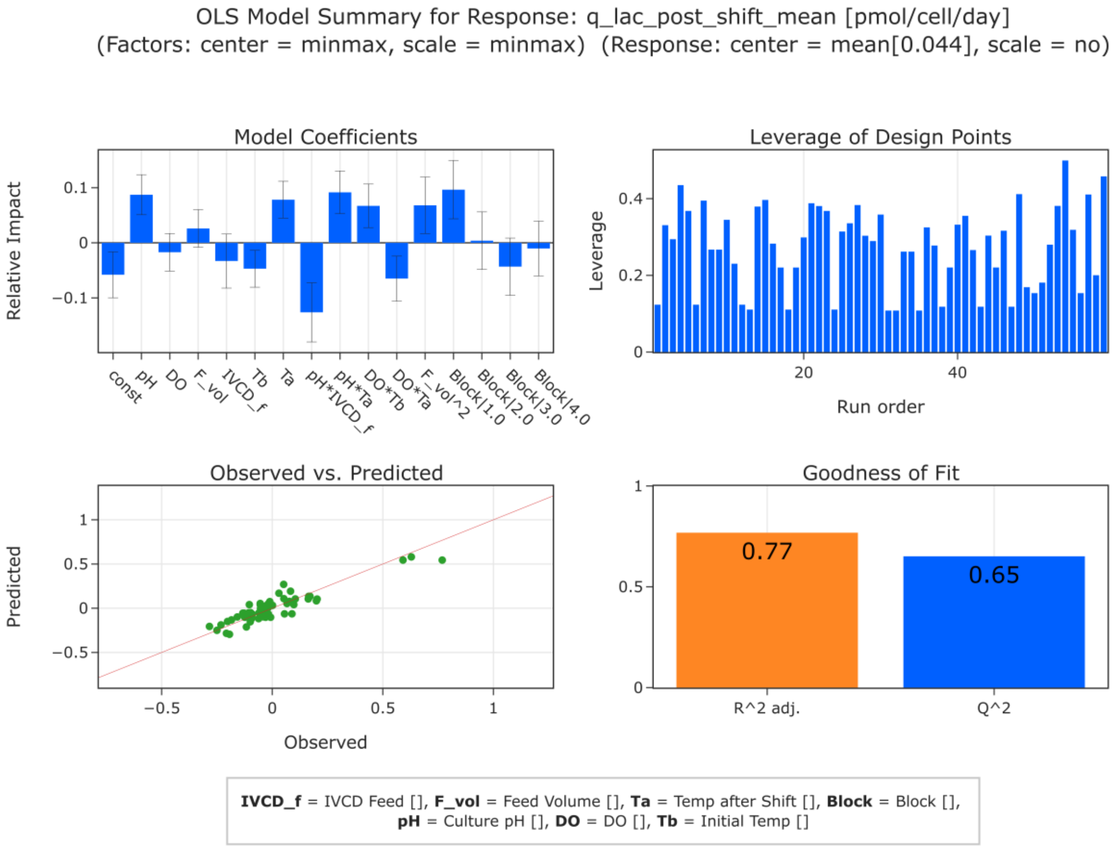

Figure S7. Model summary for the mean specific lactate conversion rate after the temperature shift. In the subplot “Model coefficients”, the scaled and centered model coefficients are shown. Main effects can be interpreted as the change of the response variable (in original units) within half the screening range of the respective factor. The coefficients of the fixed effects are depicted in blue. Displayed intervals represent the 95% confidence interval. The second subplot shows the leverage of each individual run in the model. No individual run shows particularly high leverage. In the third subplot, the observed vs predicted values by the model are displayed. In the last subplot, "Goodness of Fit", the R2 adj. and Q2 values from the model are displayed.

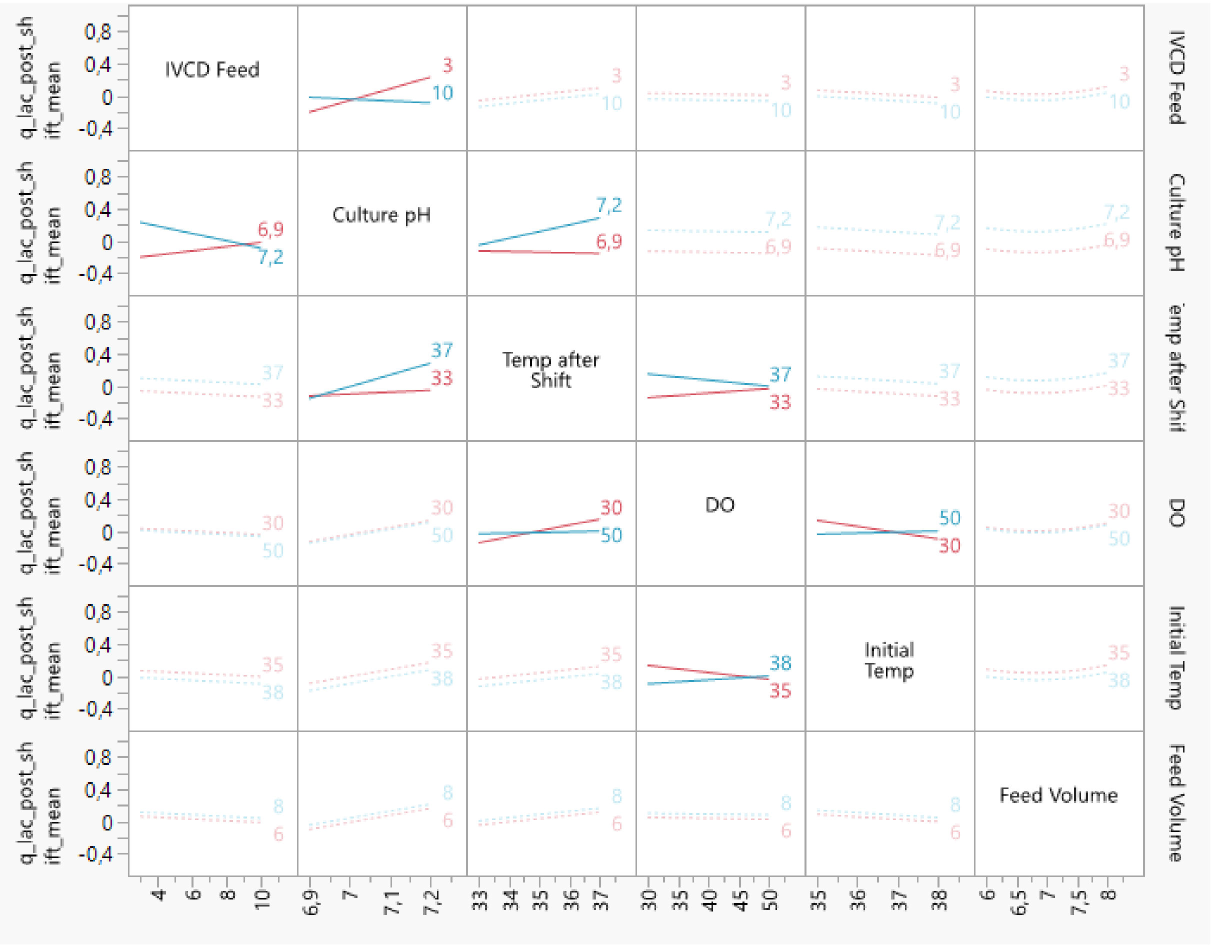

Figure S8. Interactions plot for the model describing the specific lactate conversion rate after the temperature shift.

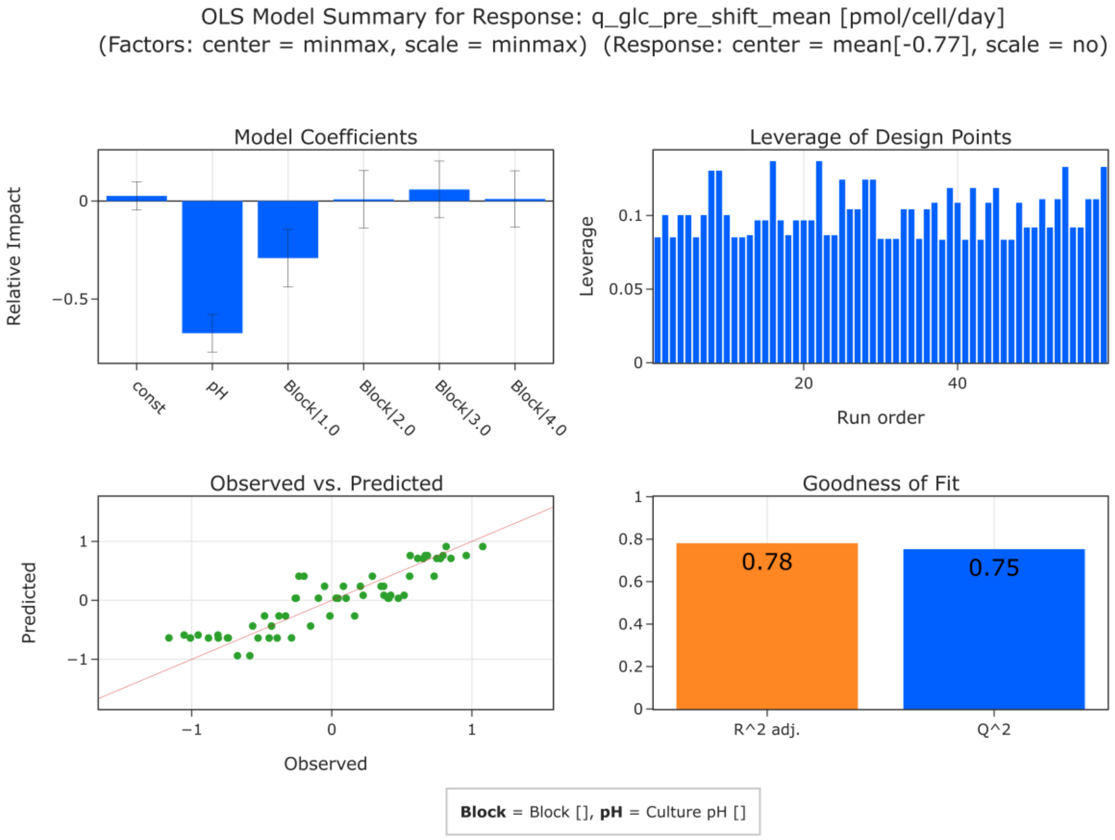

Figure S9. Model summary for the mean specific glucose uptake rate before the temperature shift. In the subplot "Model coefficients", the scaled and centered model coefficients are shown. Main effects can be interpreted as the change of the response variable (in original units) within half the screening range of the respective factor. The coefficients of the fixed effects are depicted in blue. Displayed intervals represent the 95% confidence interval. The second subplot shows the leverage of each individual run in the model. No individual run shows particularly high leverage. In the third subplot, the observed vs predicted values by the model are displayed. In the last subplot, "Goodness of Fit", the R2 adj. and Q2 values from the model are displayed.

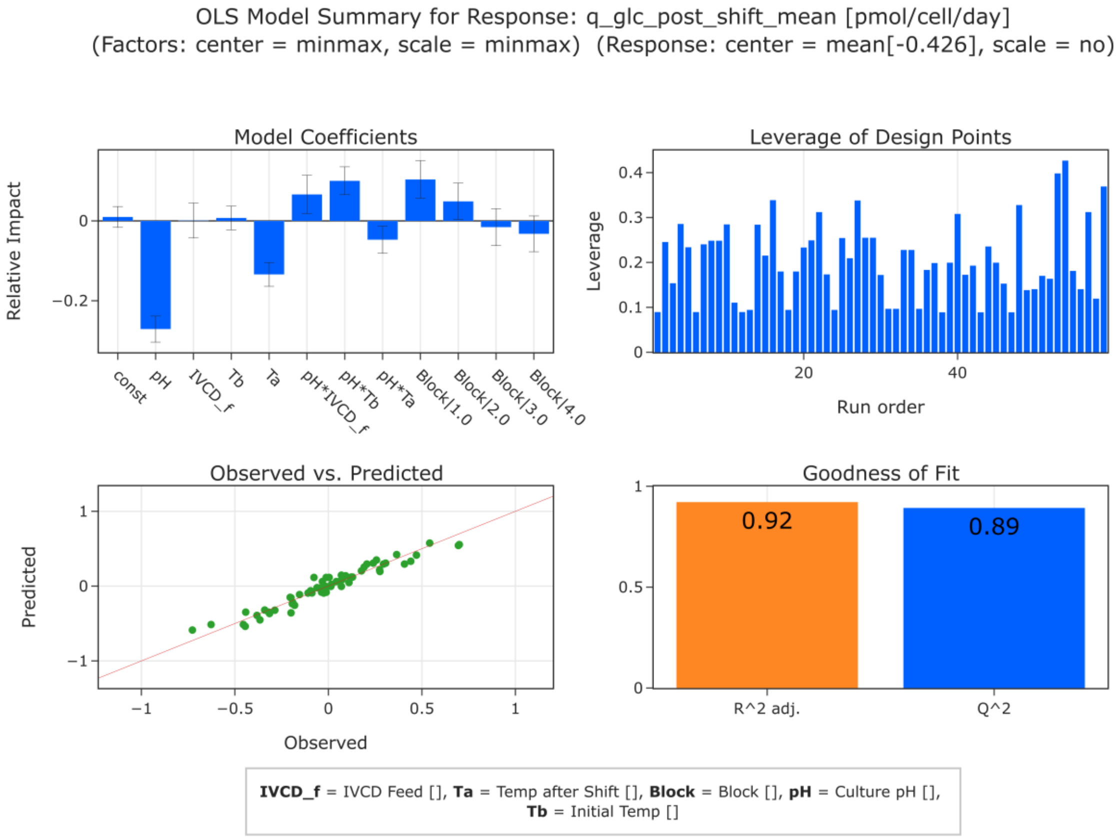

Figure S10. Model summary for the mean specific glucose uptake rate after the temperature shift. In the subplot “Model coefficients”, the scaled and centered model coefficients are shown. Main effects can be interpreted as the change of the response variable (in original units) within half the screening range of the respective factor. The coefficients of the fixed effects are depicted in blue. Displayed intervals represent the 95% confidence interval. The second subplot shows the leverage of each individual run in the model. No individual run shows particularly high leverage. In the third subplot, the observed vs predicted values by the model are displayed. In the last subplot, "Goodness of Fit", the R2 adj. and Q2 values from the model are displayed.

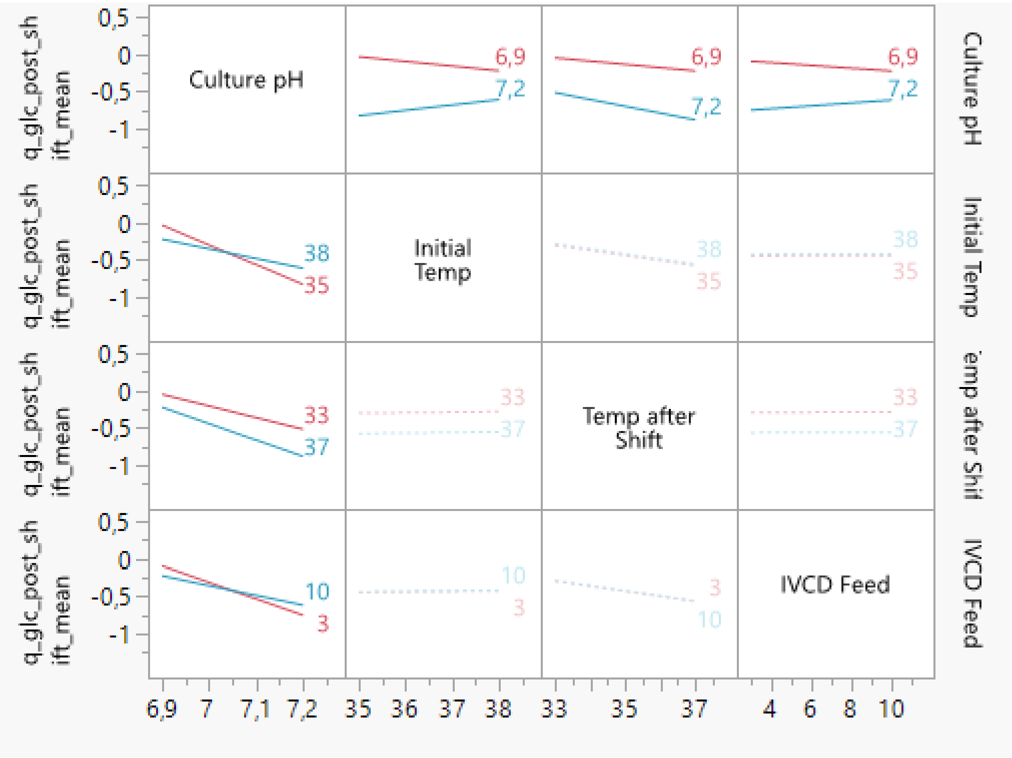

Figure S11. Interactions plot for the model describing the specific glucose uptake rate after the temperature shift.

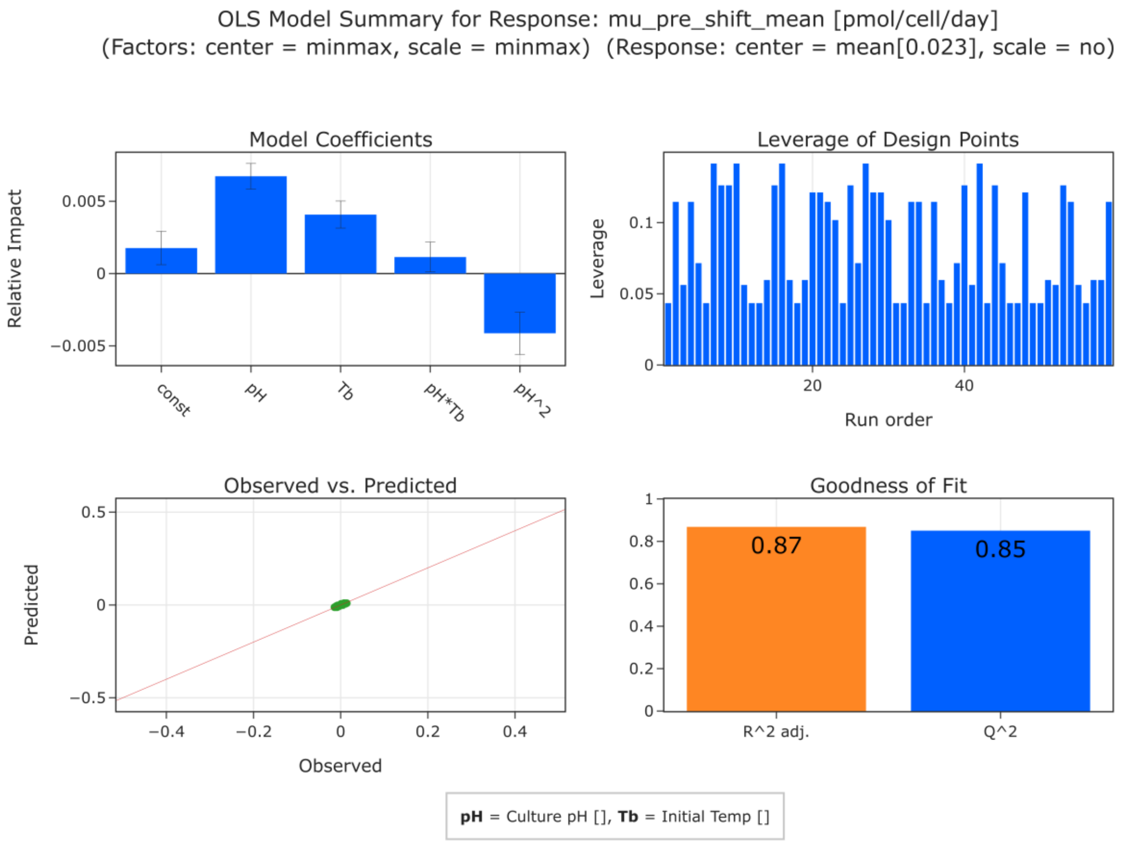

Figure S12. Model summary for the mean specific growth rate before the temperature shift. In the subplot "Model coefficients", the scaled and centered model coefficients are shown. Main effects can be interpreted as the change of the response variable (in original units) within half the screening range of the respective factor. The coefficients of the fixed effects are depicted in blue. Displayed intervals represent the 95% confidence interval. The second subplot shows the leverage of each individual run in the model. No individual run shows particularly high leverage. In the third subplot, the observed vs predicted values by the model are displayed. In the last subplot, "Goodness of Fit", the R2 adj. and Q2 values from the model are displayed.

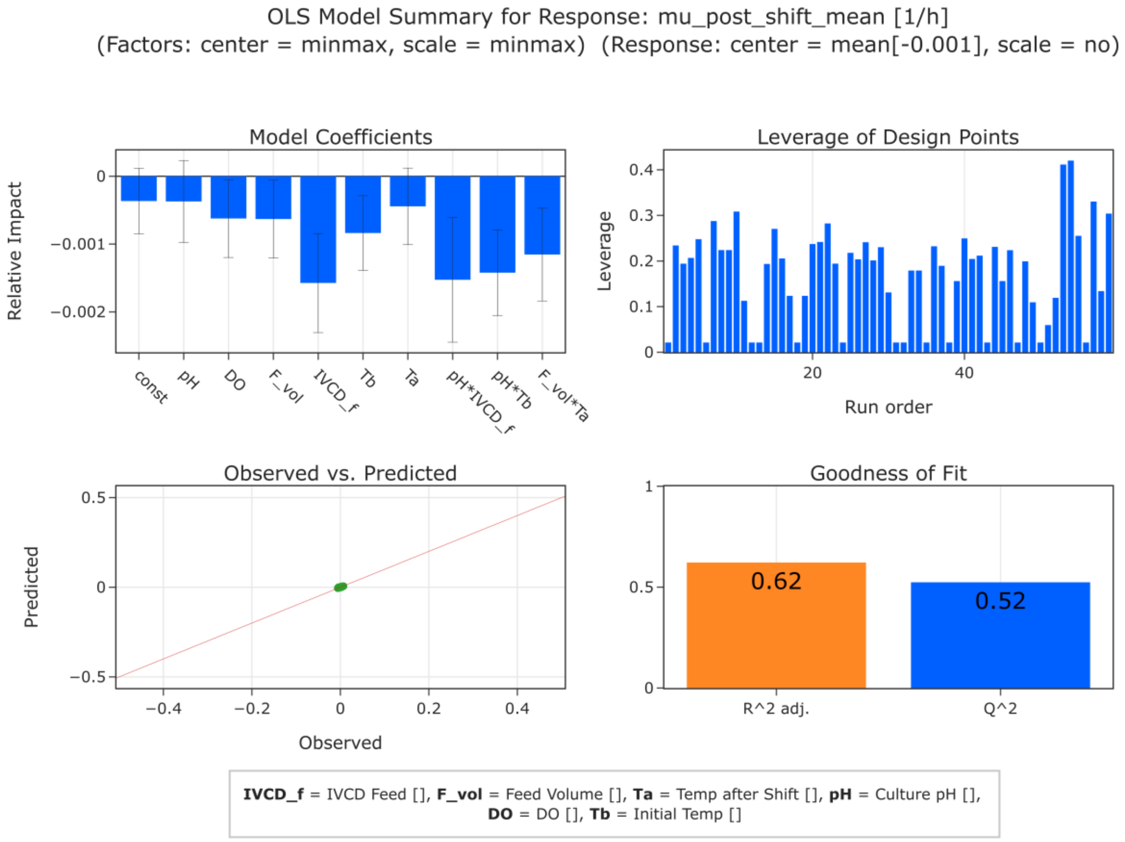

Figure S13. Model summary for the mean specific growth rate after the temperature shift. In the subplot "Model coefficients", the scaled and centered model coefficients are shown. Main effects can be interpreted as the change of the response variable (in original units) within half the screening range of the respective factor. The coefficients of the fixed effects are depicted in blue. Displayed intervals represent the 95% confidence interval. The second subplot shows the leverage of each individual run in the model. No individual run shows particularly high leverage. In the third subplot, the observed vs predicted values by the model are displayed. In the last subplot, "Goodness of Fit", the R2 adj. and Q2 values from the model are displayed.

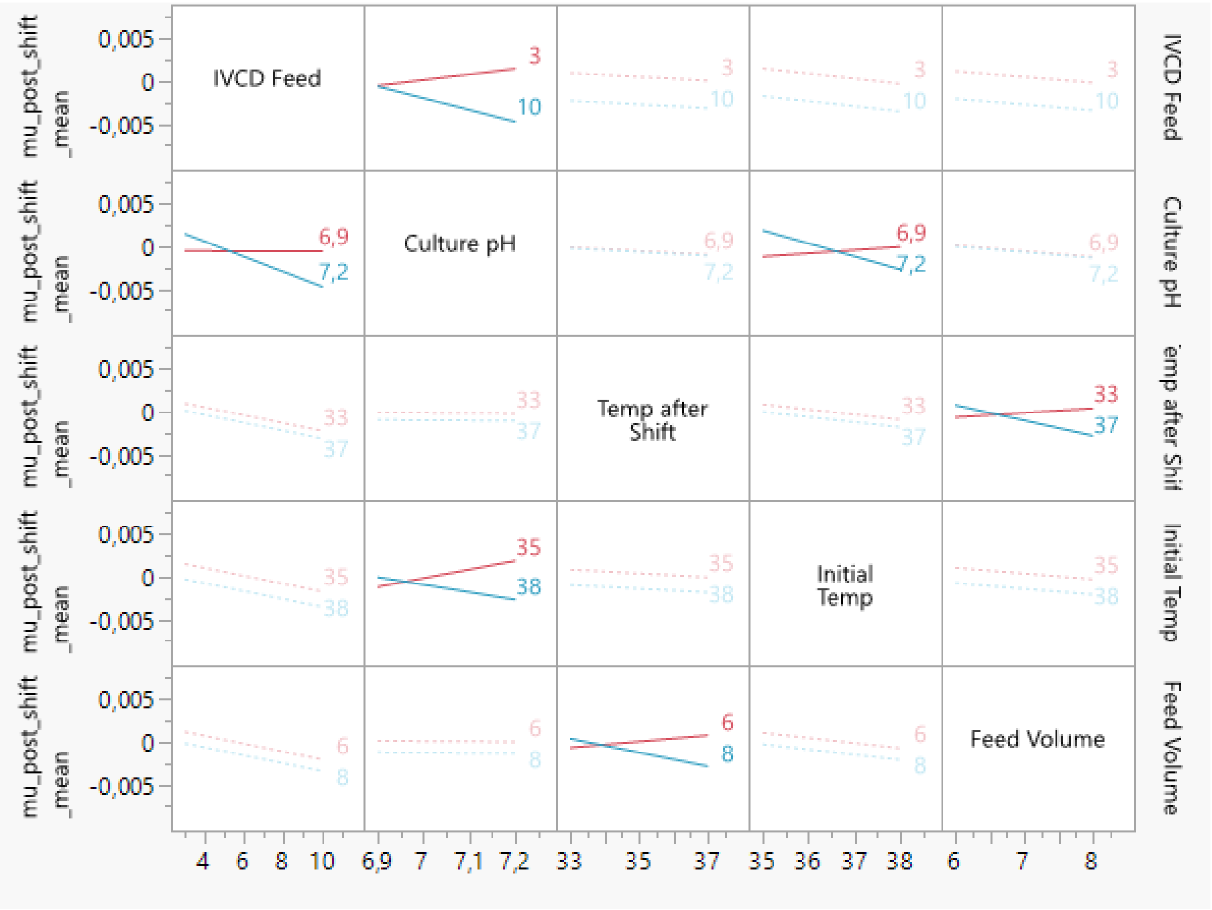

Figure S14. Interactions plot for the model describing the specific growth rate after the temperature shift.

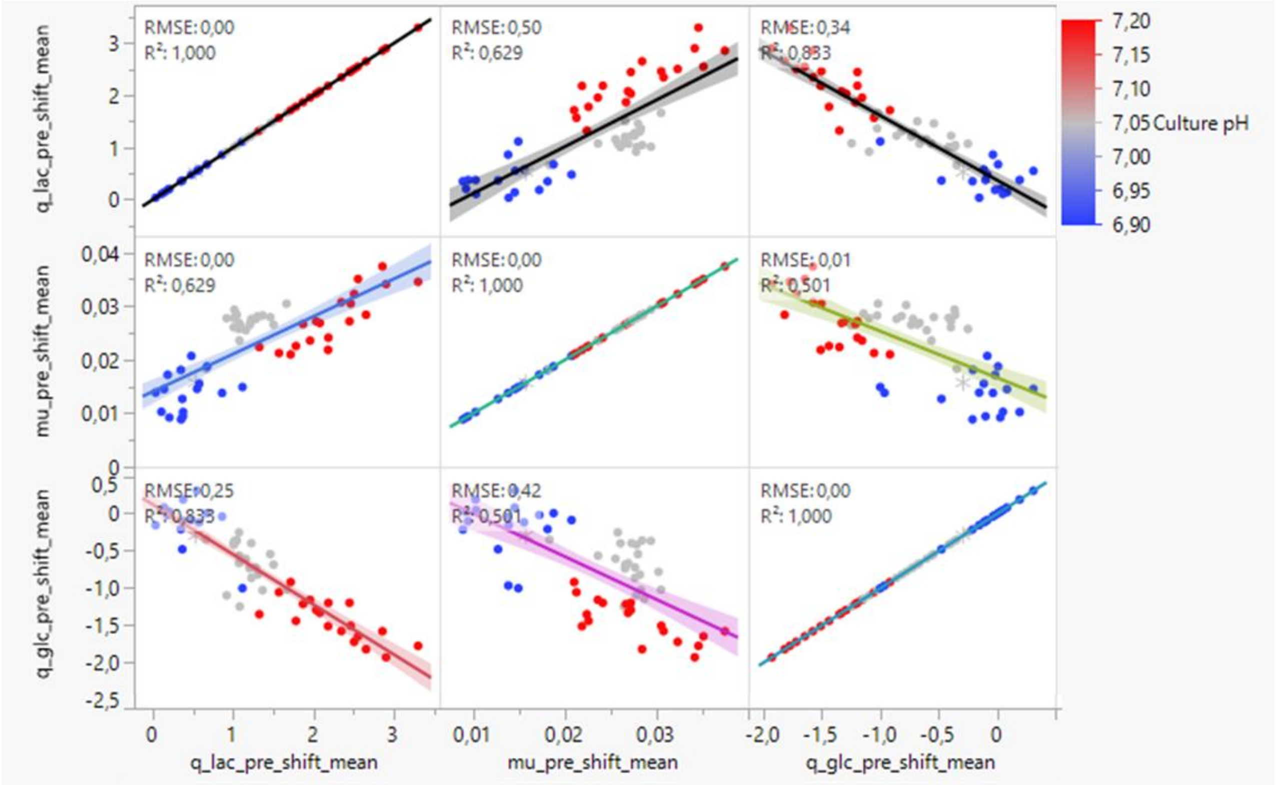

Figure S15. Cross correlation of mean of physiological rates in the phase before temperature shift.

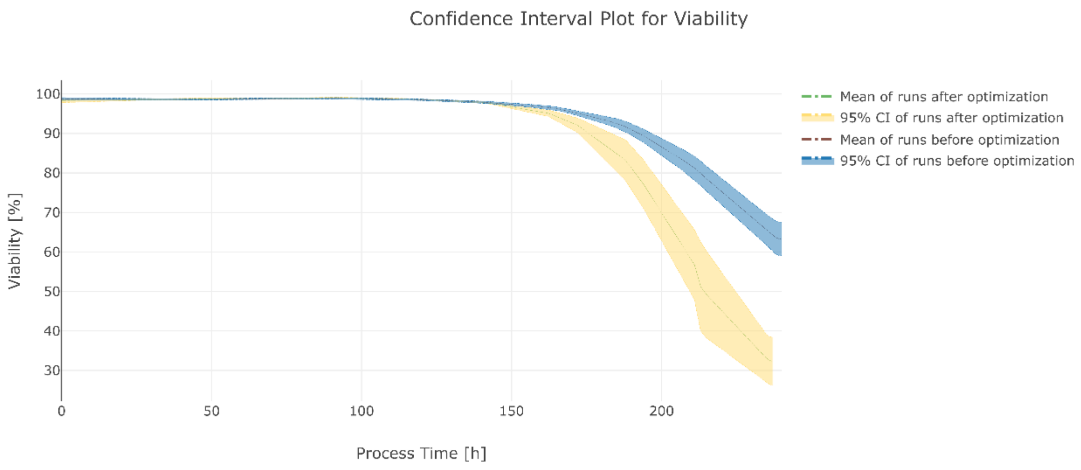

Figure S16. Comparison of the viability trend over time before and after optimization. The mean and 95% confidence interval of 15 small scale runs before optimization is shown in green and yellow. The mean and 95% confidence interval of 16 small scale runs after optimization is shown in brown and blue.

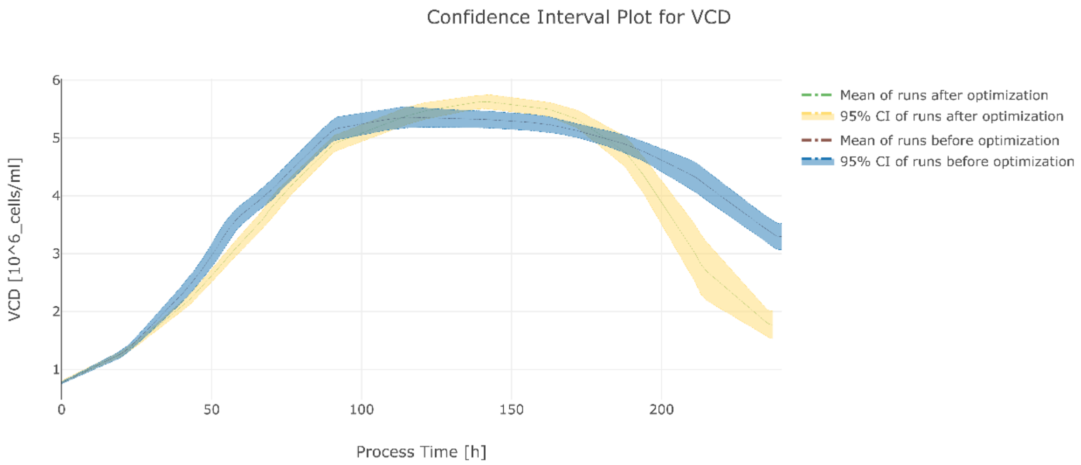

Figure S17. Comparison of the VCD trend over time before and after optimization. The mean and 95% confidence interval of 15 small scale runs before optimization is shown in green and yellow. The mean and 95% confidence interval of 16 small scale runs after optimization is shown in brown and blue.

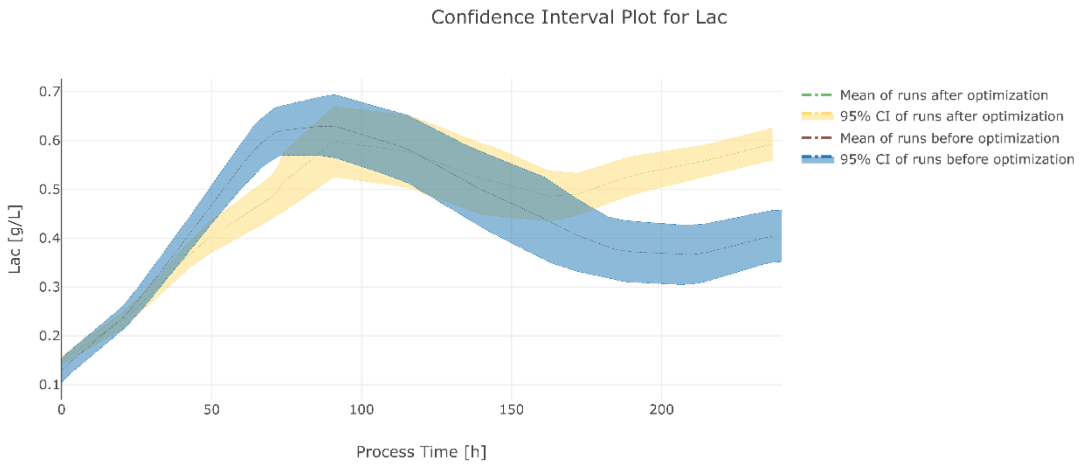

Figure S18. Comparison of the lactate trend over time before and after optimization. The mean and 95% confidence interval of 15 small scale runs before optimization is shown in green and yellow. The mean and 95% confidence interval of 16 small scale runs after optimization is shown in brown and blue.

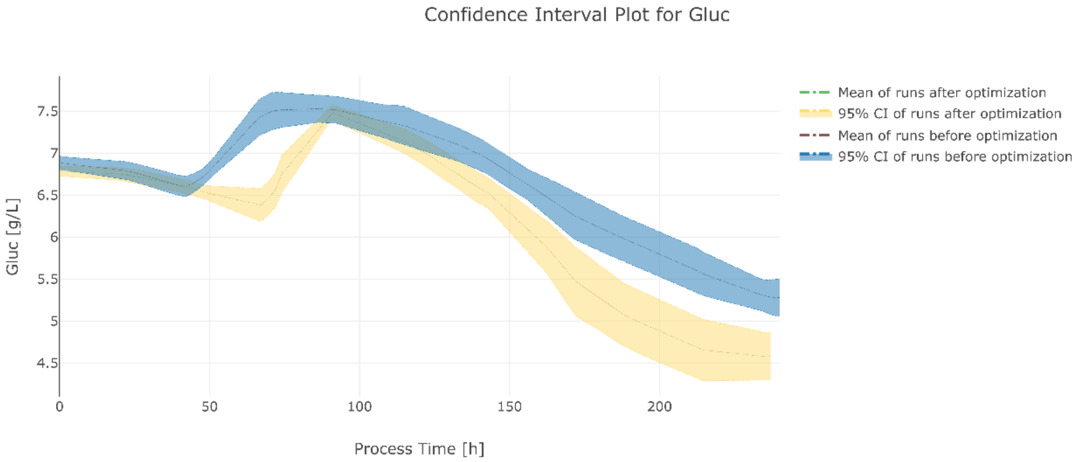

Figure S19. Comparison of the glucose trend over time before and after optimization. The mean and 95% confidence interval of 15 small scale runs before optimization is shown in green and yellow. The mean and 95% confidence interval of 16 small scale runs after optimization is shown in brown and blue.

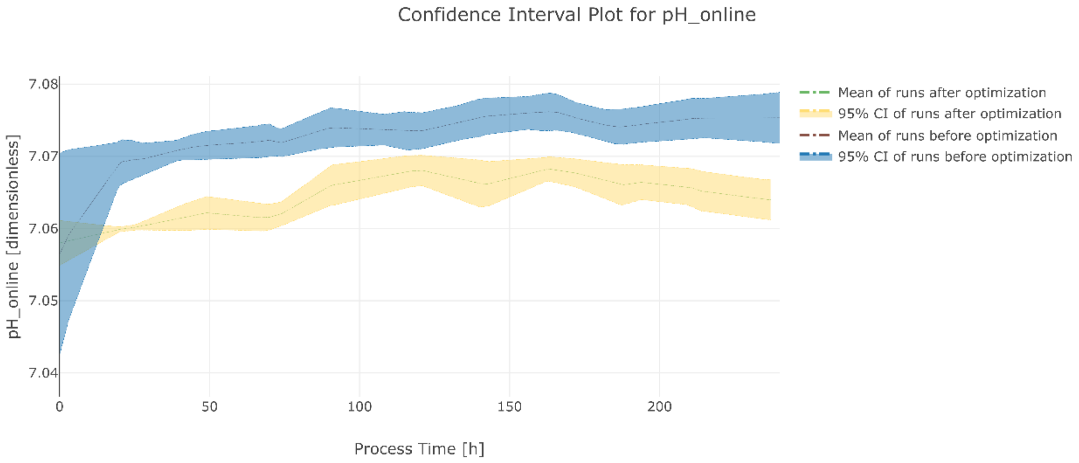

Figure S20. Comparison of the pH (measured online) trend over time before and after optimization. The mean and 95% confidence interval of 15 small scale runs before optimization is shown in green and yellow. The mean and 95% confidence interval of 16 small scale runs after optimization is shown in brown and blue.
